# Supplementary material for: Cadinapyridine sesquiterpene alkaloids from Artemisia annua and in vitro cytotoxicity and antiplasmodial activities
Source: PLoS One. 2025 Sep 3;20(9):e0331186. doi: 10.1371/journal.pone.0331186 (PMC12407398; doi:10.1371/journal.pone.0331186)
Supplement: S1 File — (DOCX) [file pone.0331186.s001.docx]

**Supplementary Materials**

**Cadinapyridine sesquiterpene alkaloids from *Artemisia annua* and *in vitro* cytotoxicity and antiplasmodial activities**

Nicolas Fabre^1*^, Adrien Vitrai^1^, Sandra Bourgeade-Delmas^1^, Nathalie Saffon-Merceron^2^, Abdou Madjid Olatoundé Amoussa^3^, Latifou Lagnika^3^, Agnès Aubouy^1^ and Valérie Jullian^1^.

^1^ UMR 152 PharmaDev, Université de Toulouse, UPS, IRD, Toulouse, France

^2^ Institut de Chimie de Toulouse, ICT UAR 2599, Université Paul Sabatier-Toulouse III, Toulouse, France

^3^ Laboratoire de Biochimie et Substances Naturelles Bioactives (LBSNB), Faculté des Sciences et Techniques (FAST), Université d’Abomey Calavi, Bénin


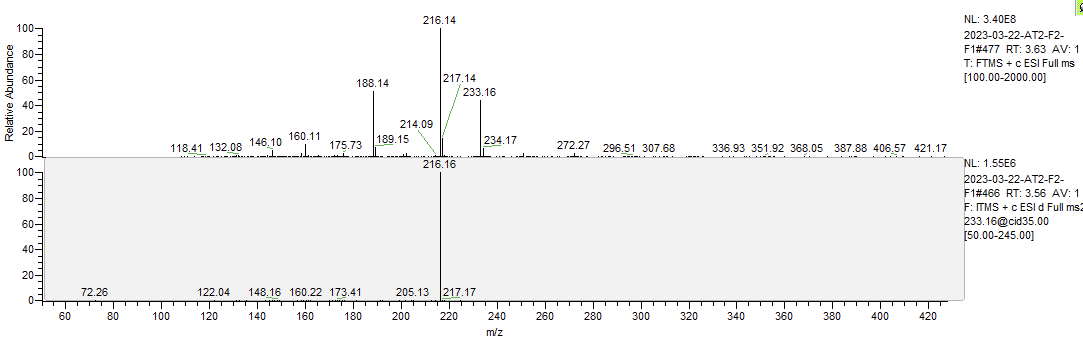


**Fig S1:** Positive ion MS and MS/MS spectra of compound **1**.


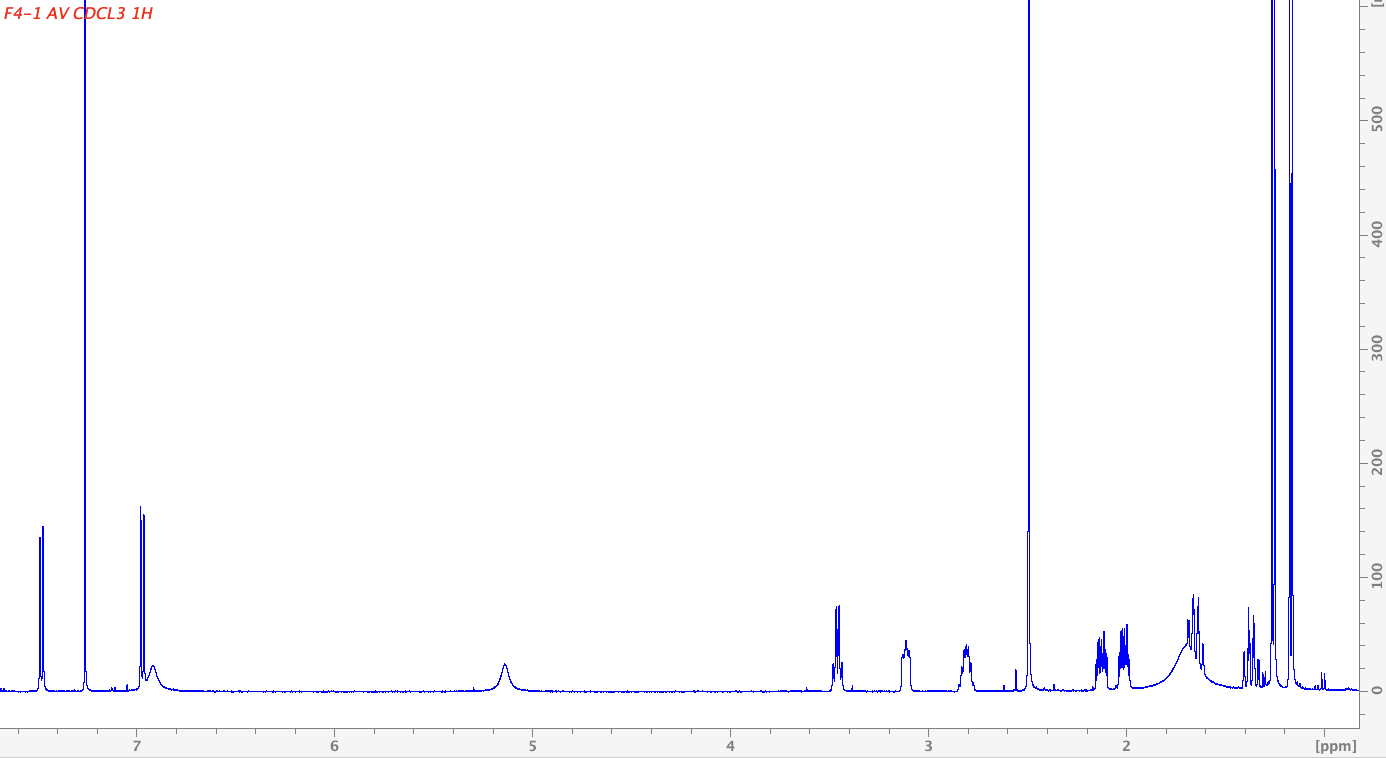

**Fig S2:** ^1^H NMR spectrum of compound **1** (500 MHz, CDCl_3_).


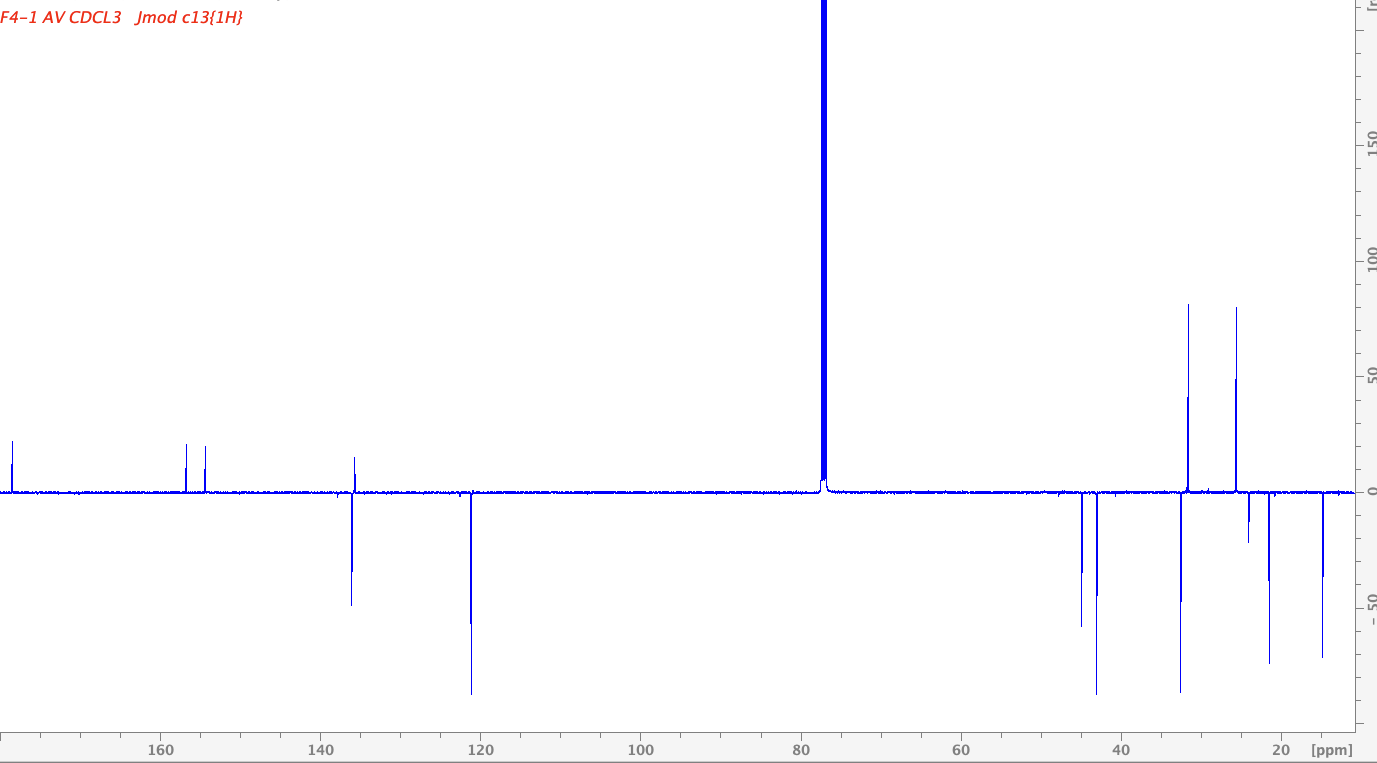


**Fig S3:** ^13^C NMR spectrum of compound **1** (125 MHz, CDCl_3_).


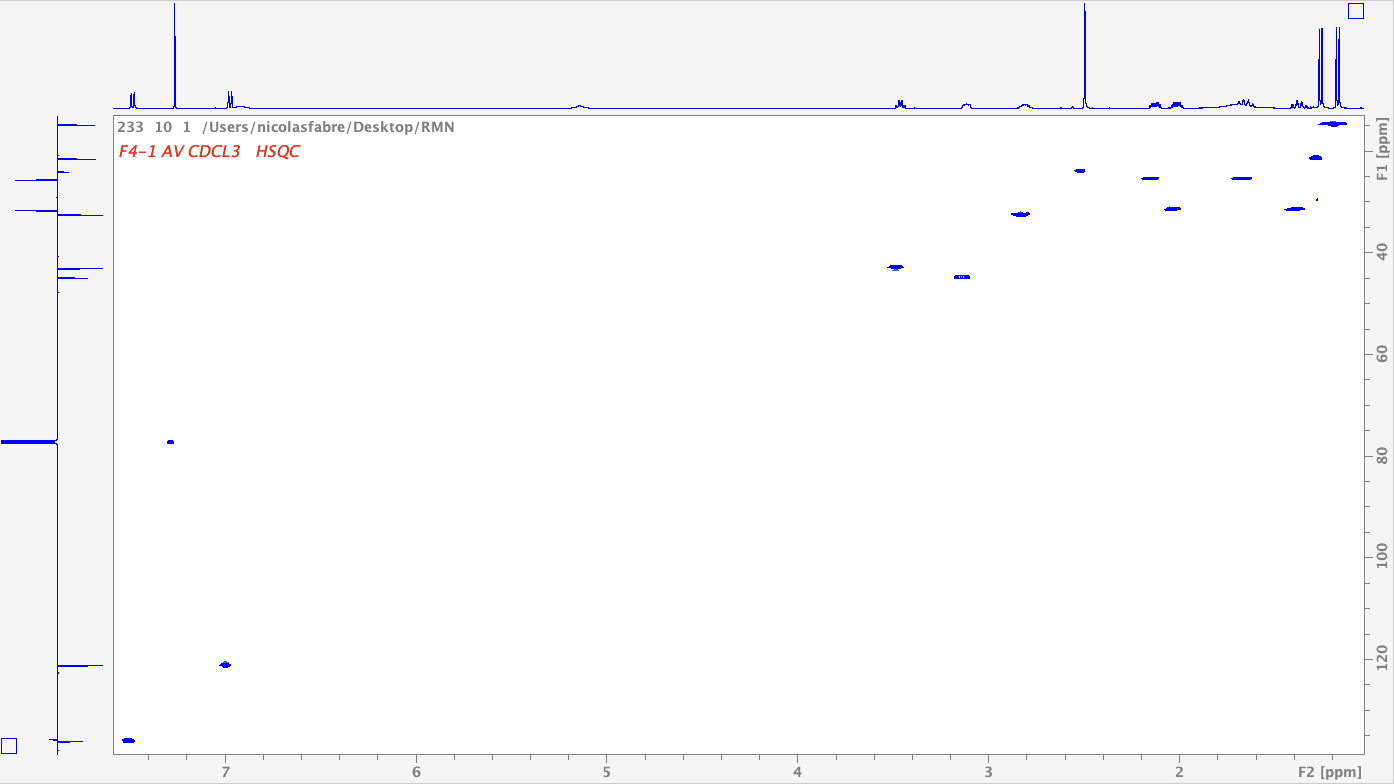


**Fig S4:** HSQC NMR spectrum of compound **1** (500 MHz, CDCl_3_).


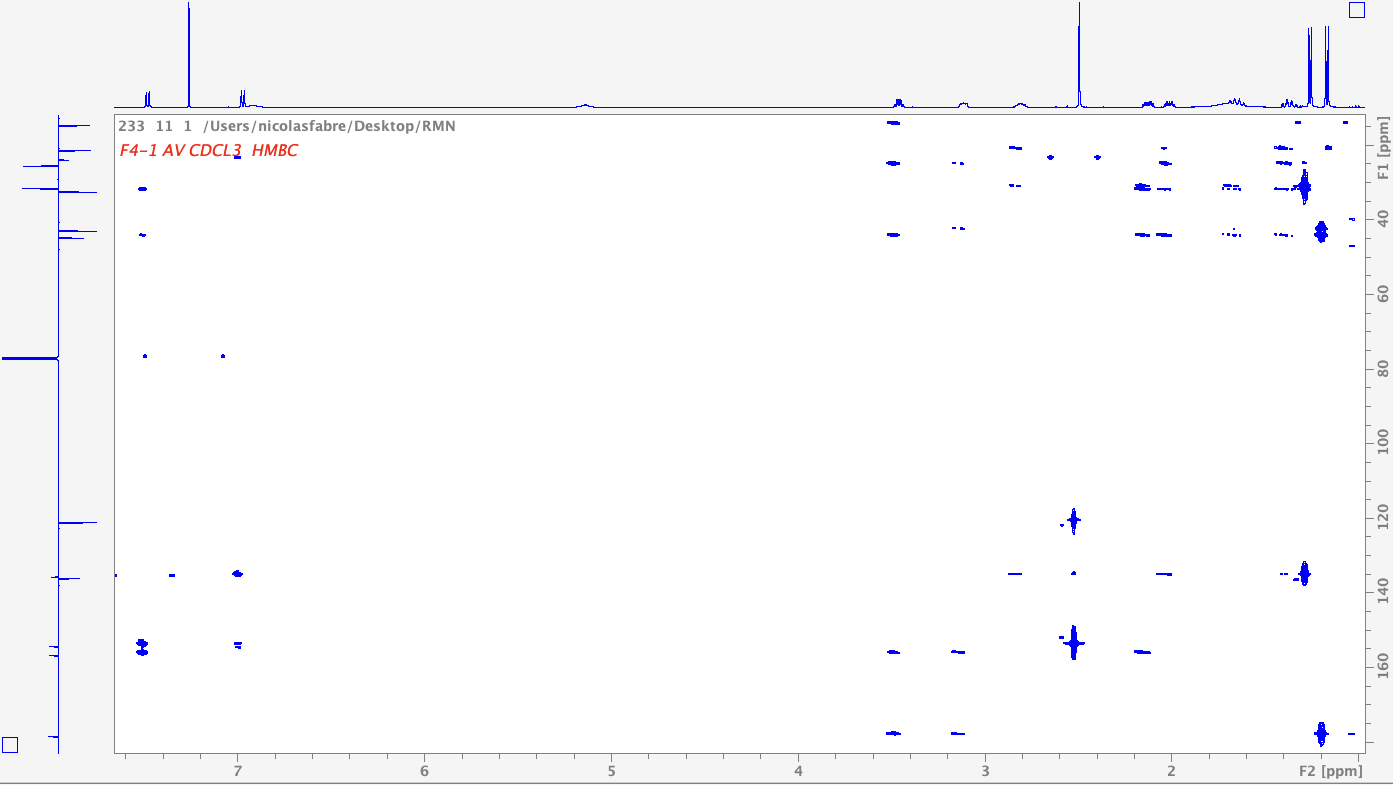


**Fig S5:** HMBC NMR spectrum of compound **1** (500 MHz, CDCl_3_).


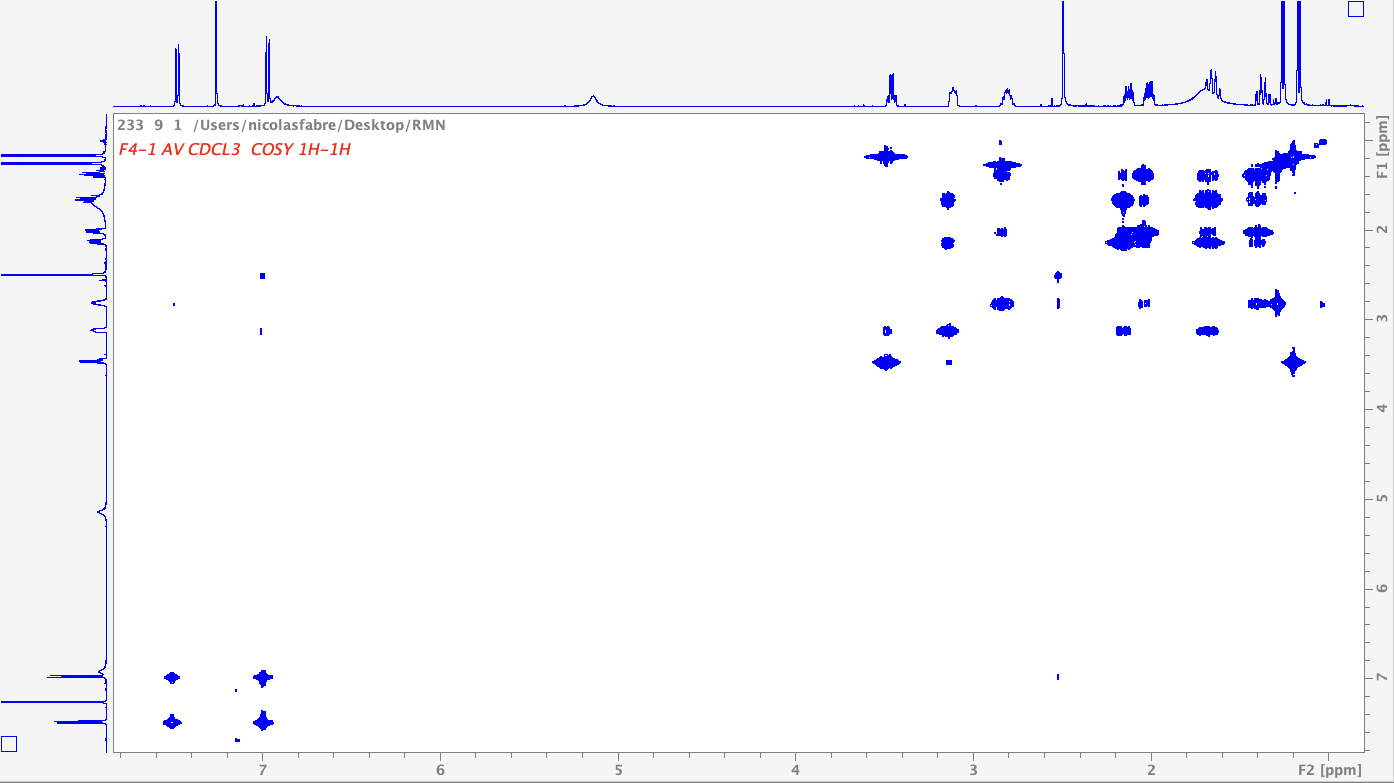


**Fig S6:** COSY NMR spectrum of compound **1** (500 MHz, CDCl_3_).


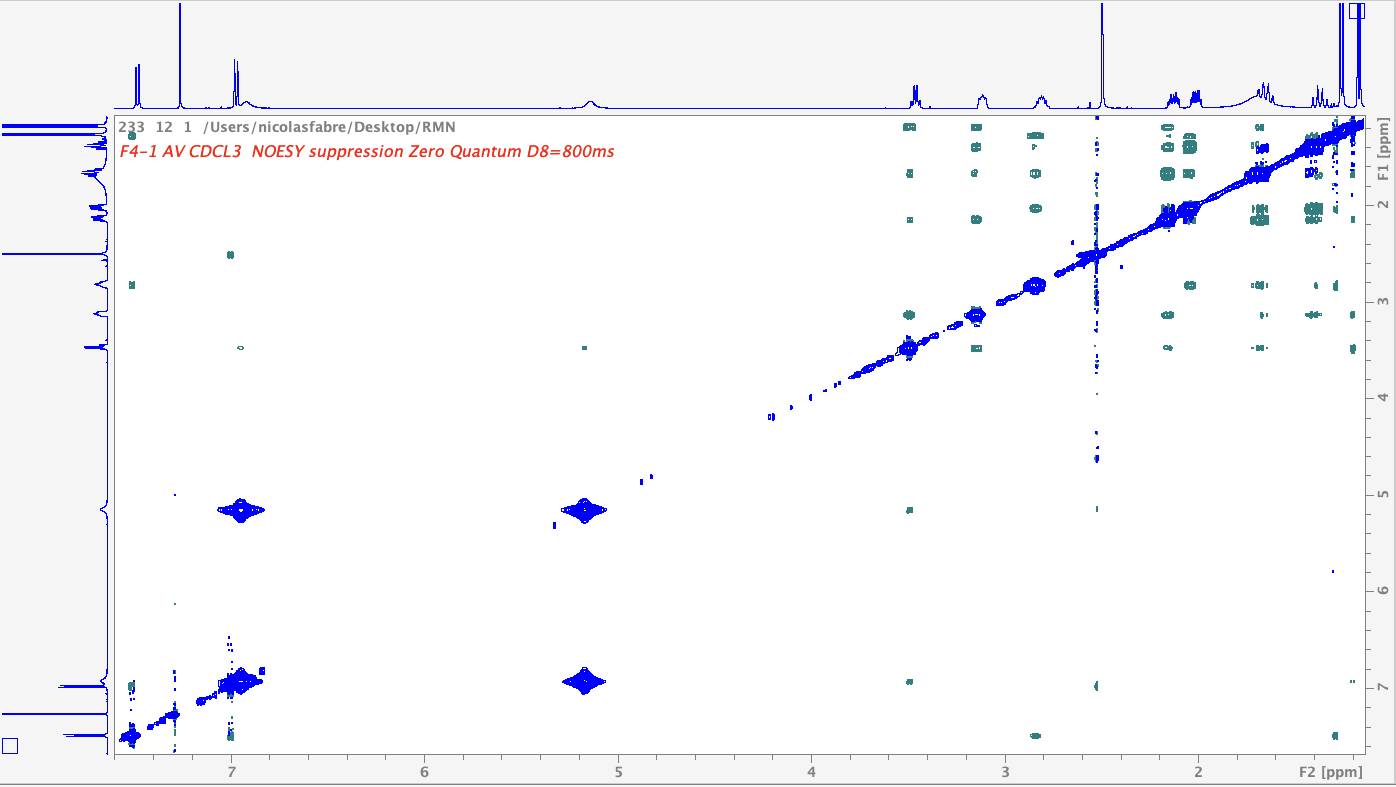


**Fig S7:** NOESY NMR spectrum of compound **1** (500 MHz, CDCl_3_).


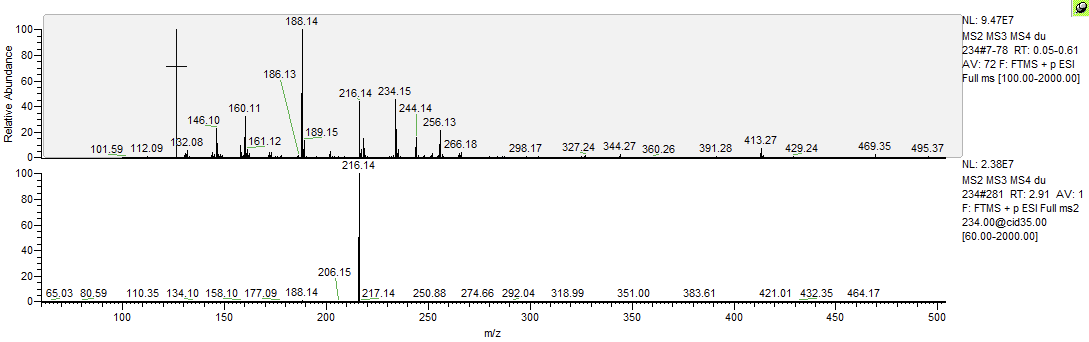


**Fig S8:** Positive ion MS and MS/MS spectra of compound **2**.


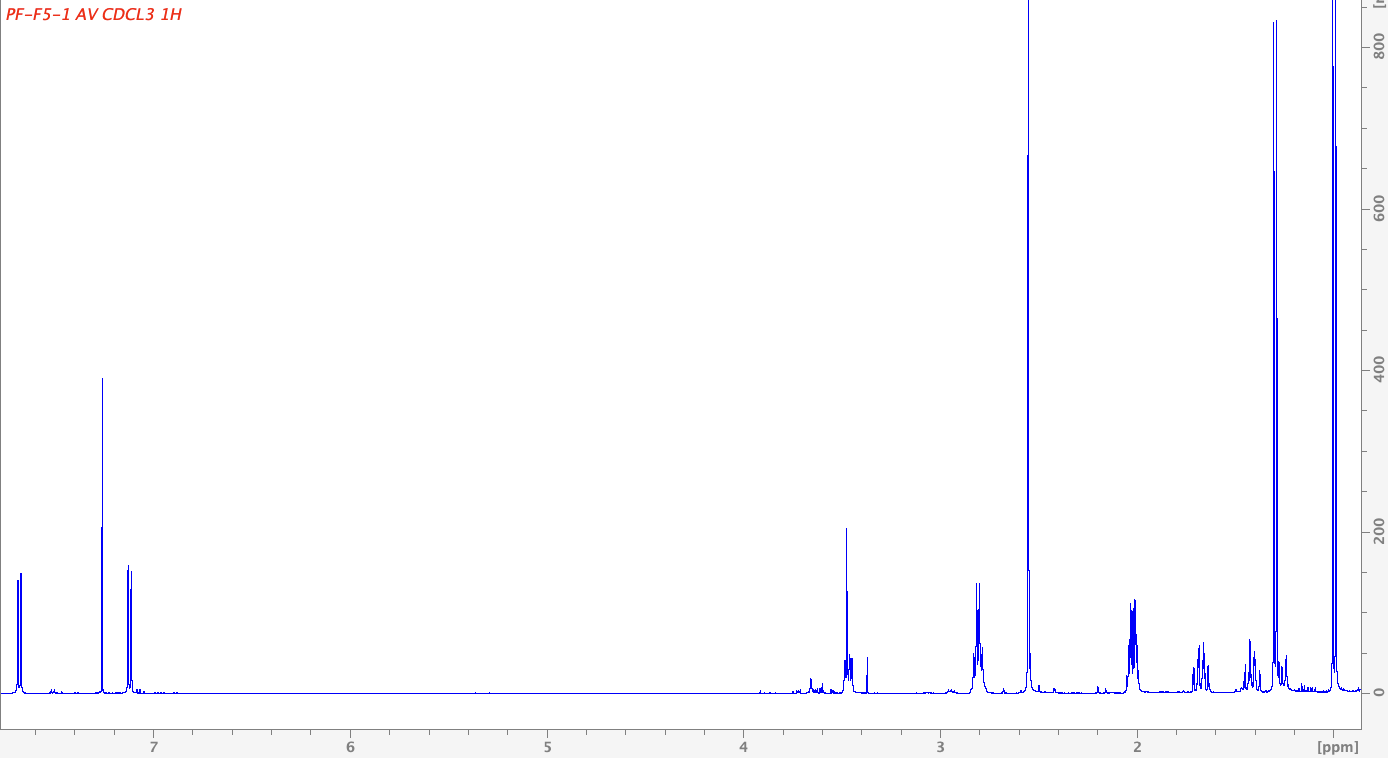

**Fig S9:** ^1^H NMR spectrum of compound **2** (500 MHz, CDCl_3_).


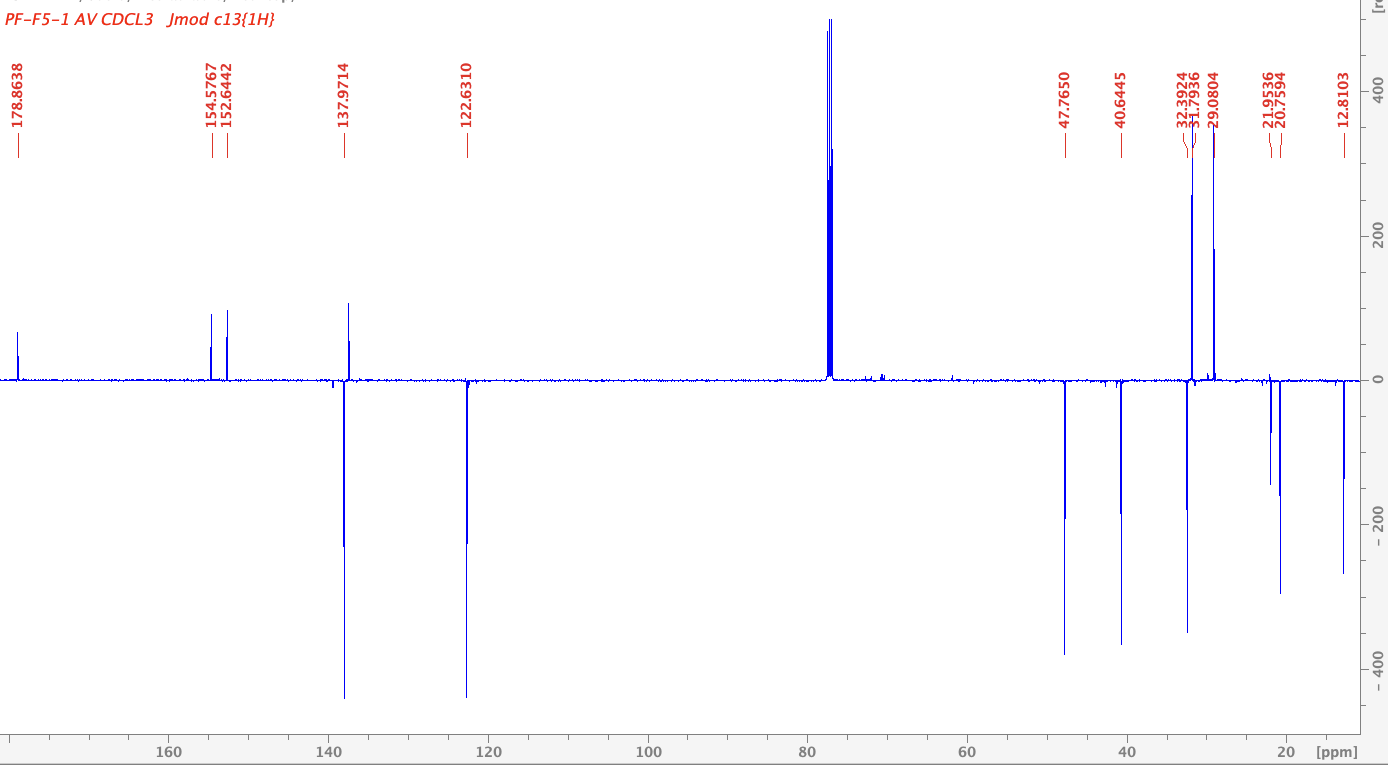


**Fig S10:** ^13^C NMR spectrum of compound **2** (125 MHz, CDCl_3_).


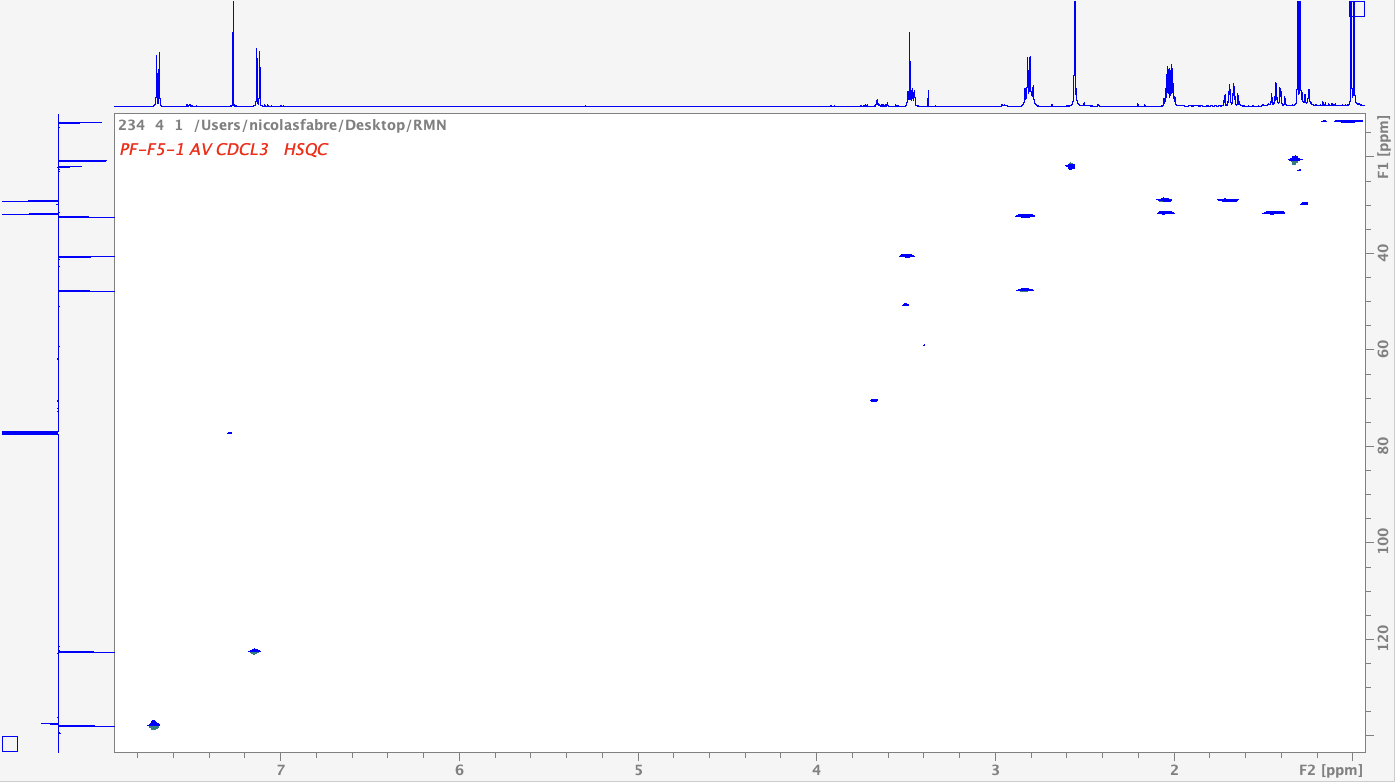


**Fig S11:** HSQC NMR spectrum of compound **2** (500 MHz, CDCl_3_).


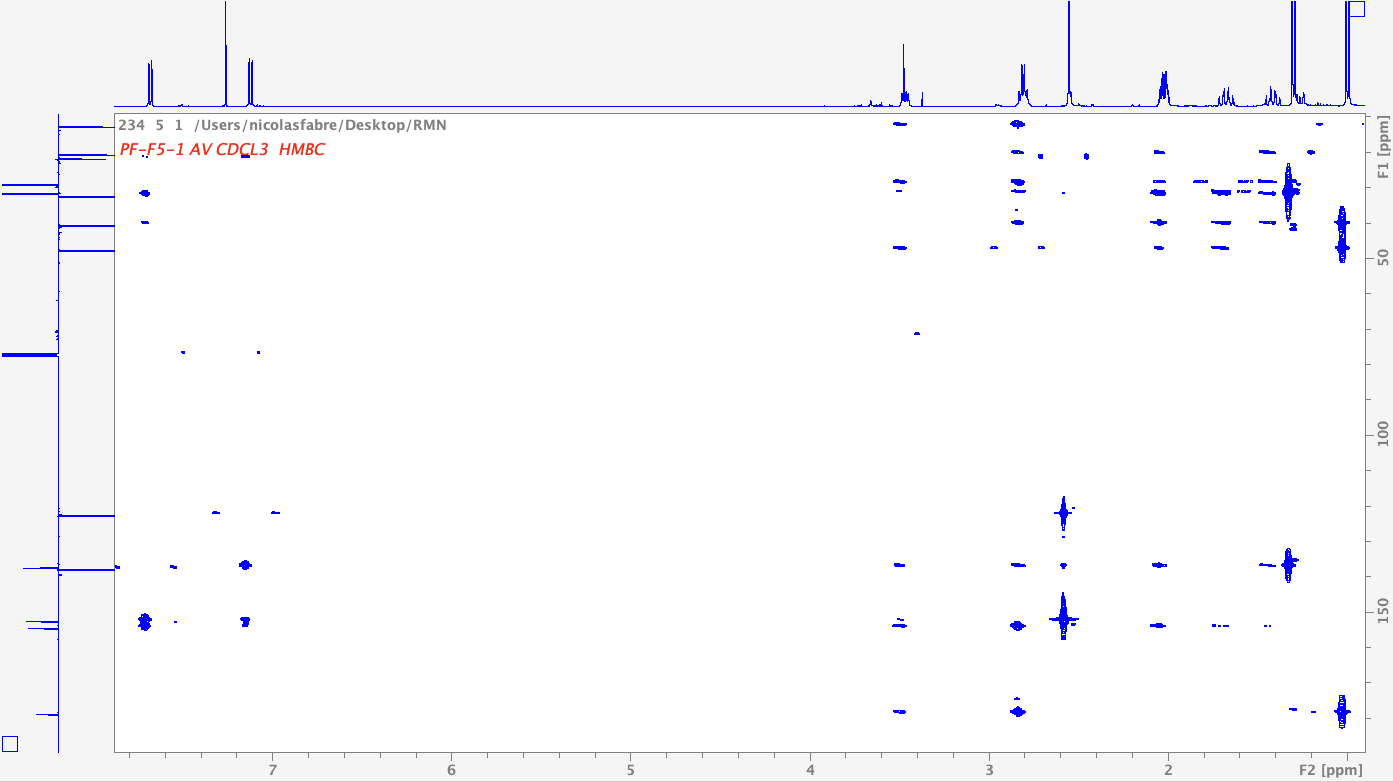


**Fig S12:** HMBC NMR spectrum of compound **2** (500 MHz, CDCl_3_).


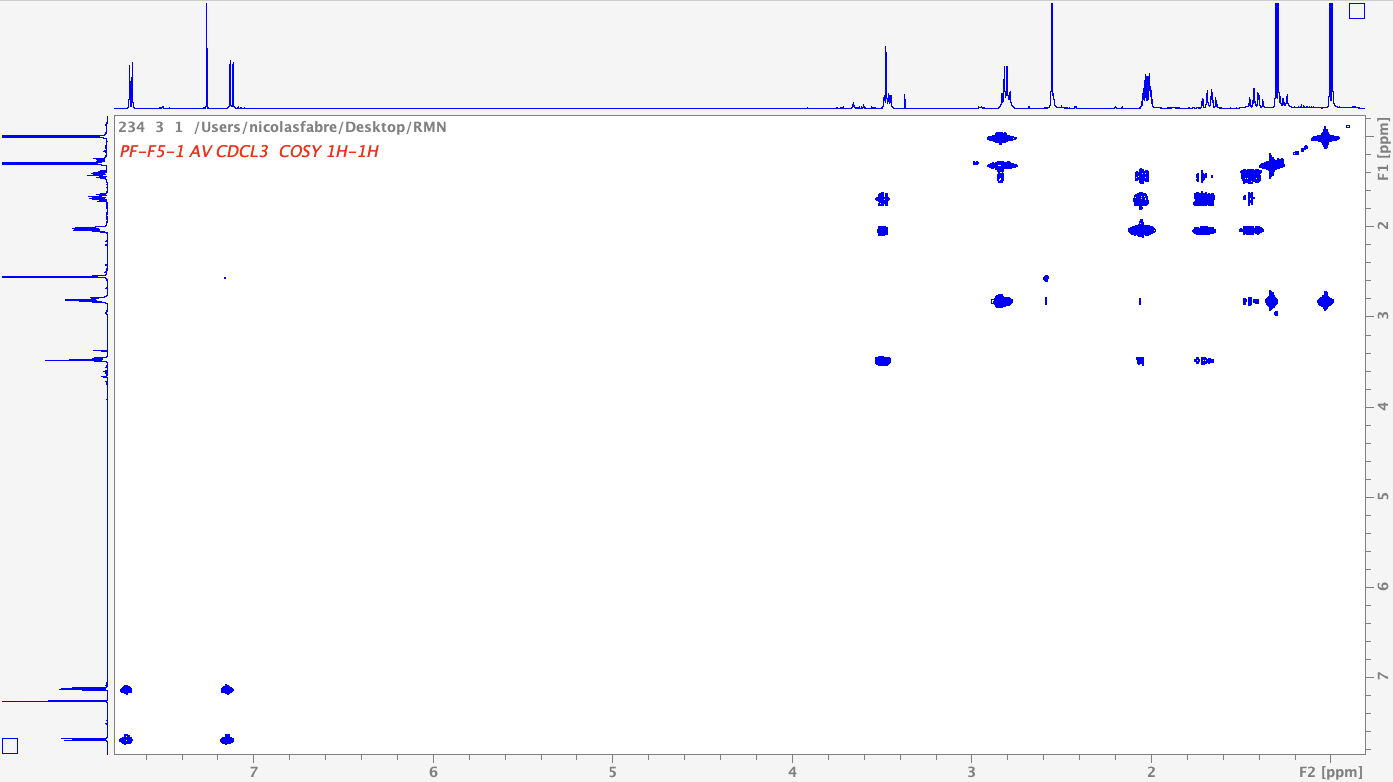


**Fig S13:** COSY NMR spectrum of compound **2** (500 MHz, CDCl_3_).


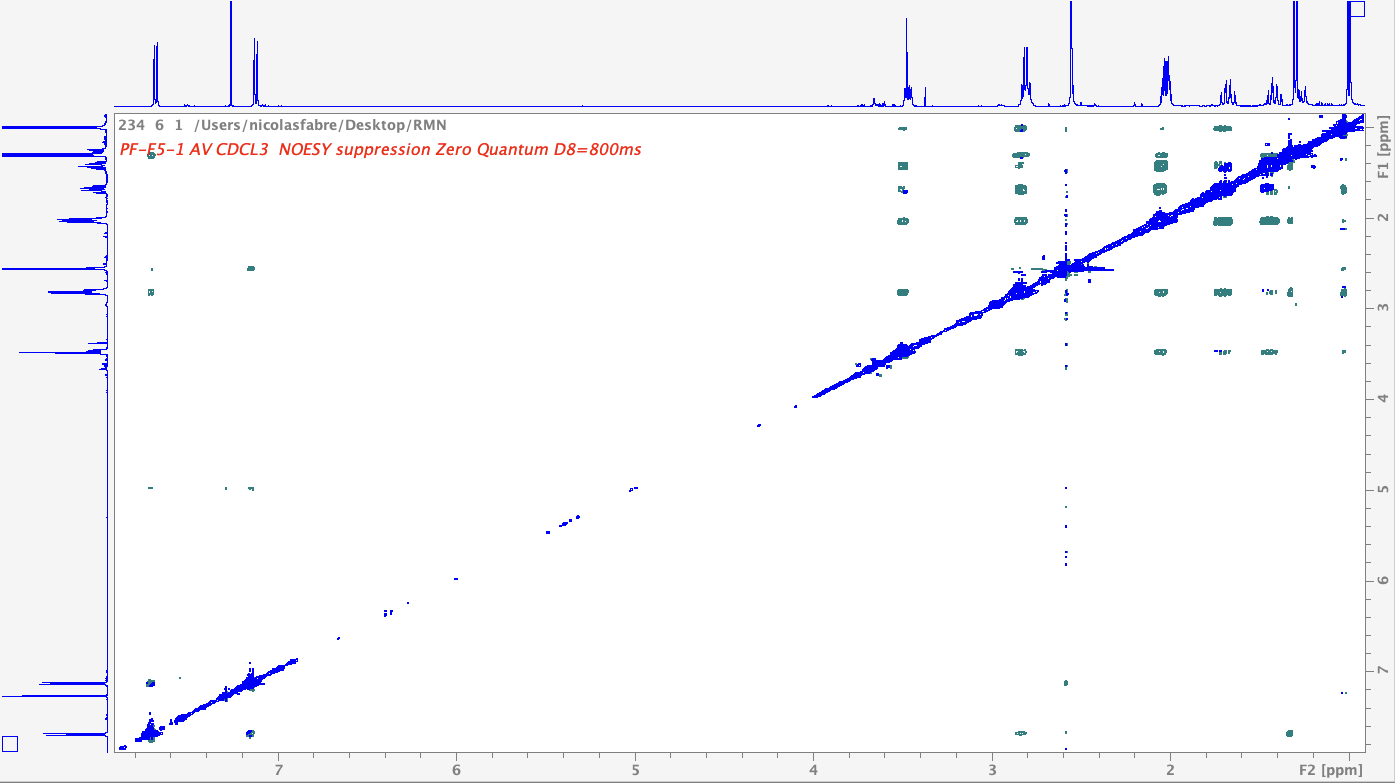


**Fig S14:** NOESY NMR spectrum of compound **2** (500 MHz, CDCl_3_).


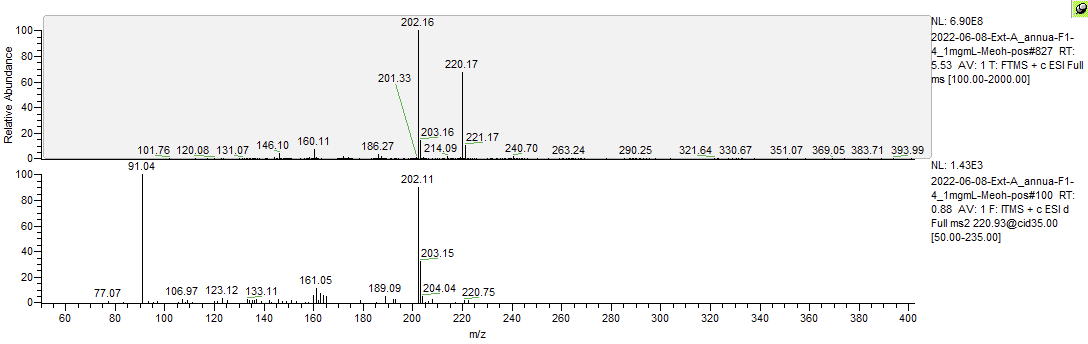


**Fig S15:** Positive ion MS and MS/MS spectra of compound **3**.


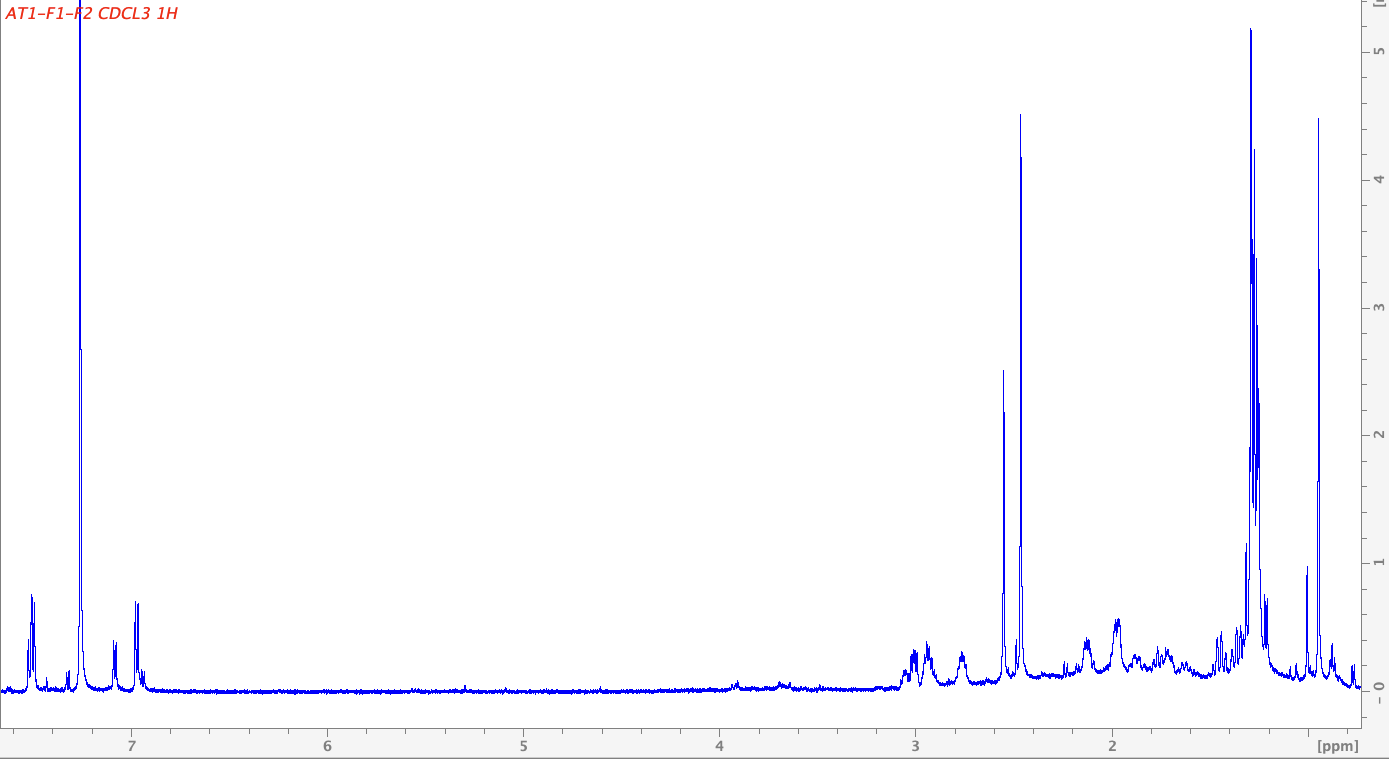

**Fig S16:** ^1^H NMR spectrum of compound **3** (500 MHz, CDCl_3_).


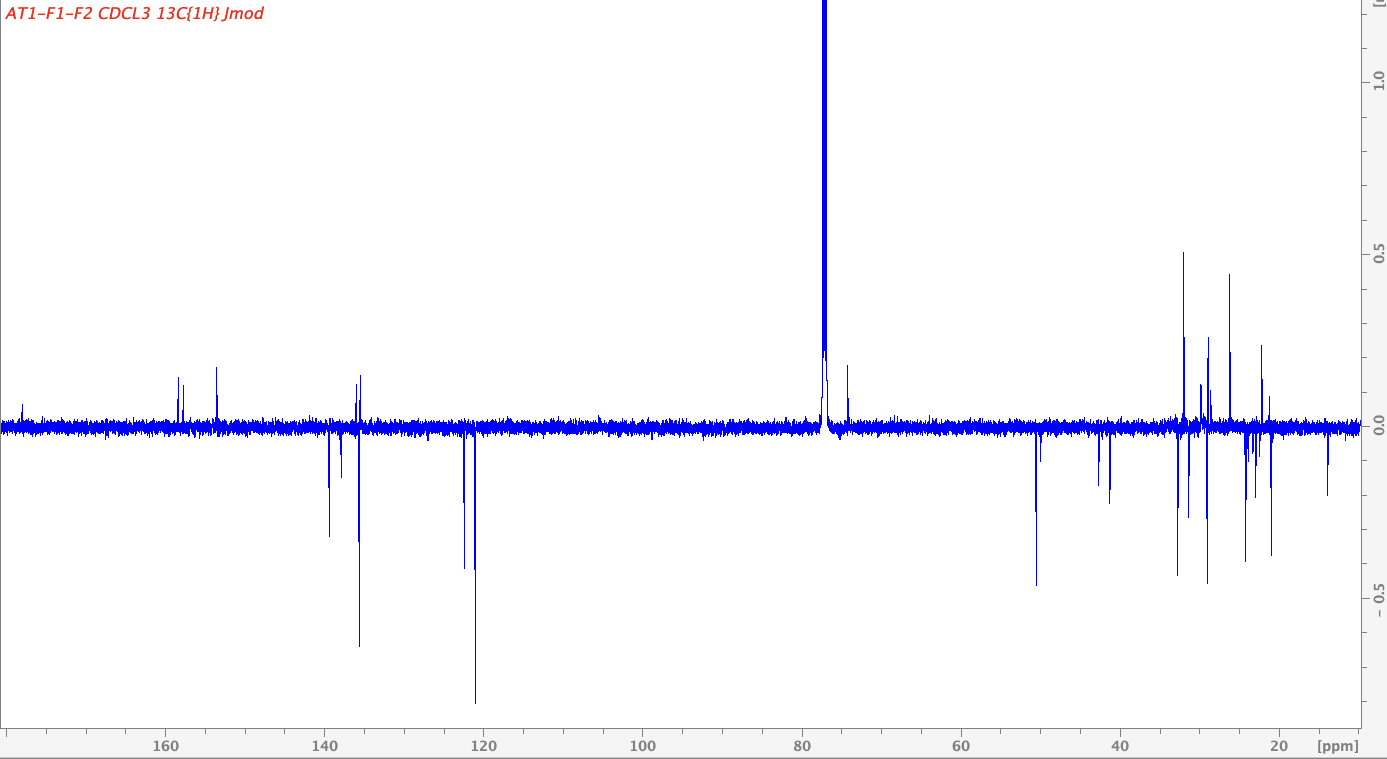


**Fig S17:** ^13^C NMR spectrum of compound **3** (125 MHz, CDCl_3_).


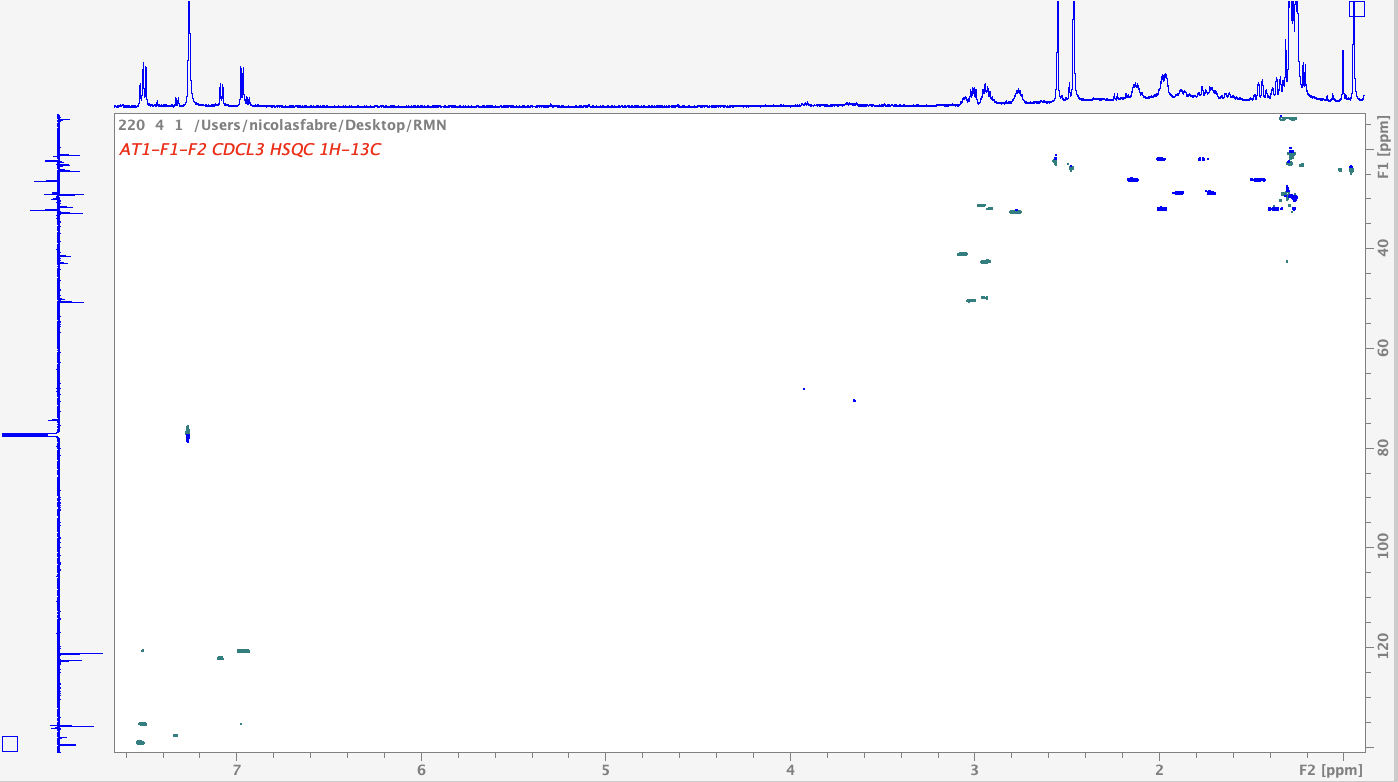


**Fig S18:** HSQC NMR spectrum of compound **3** (500 MHz, CDCl_3_).


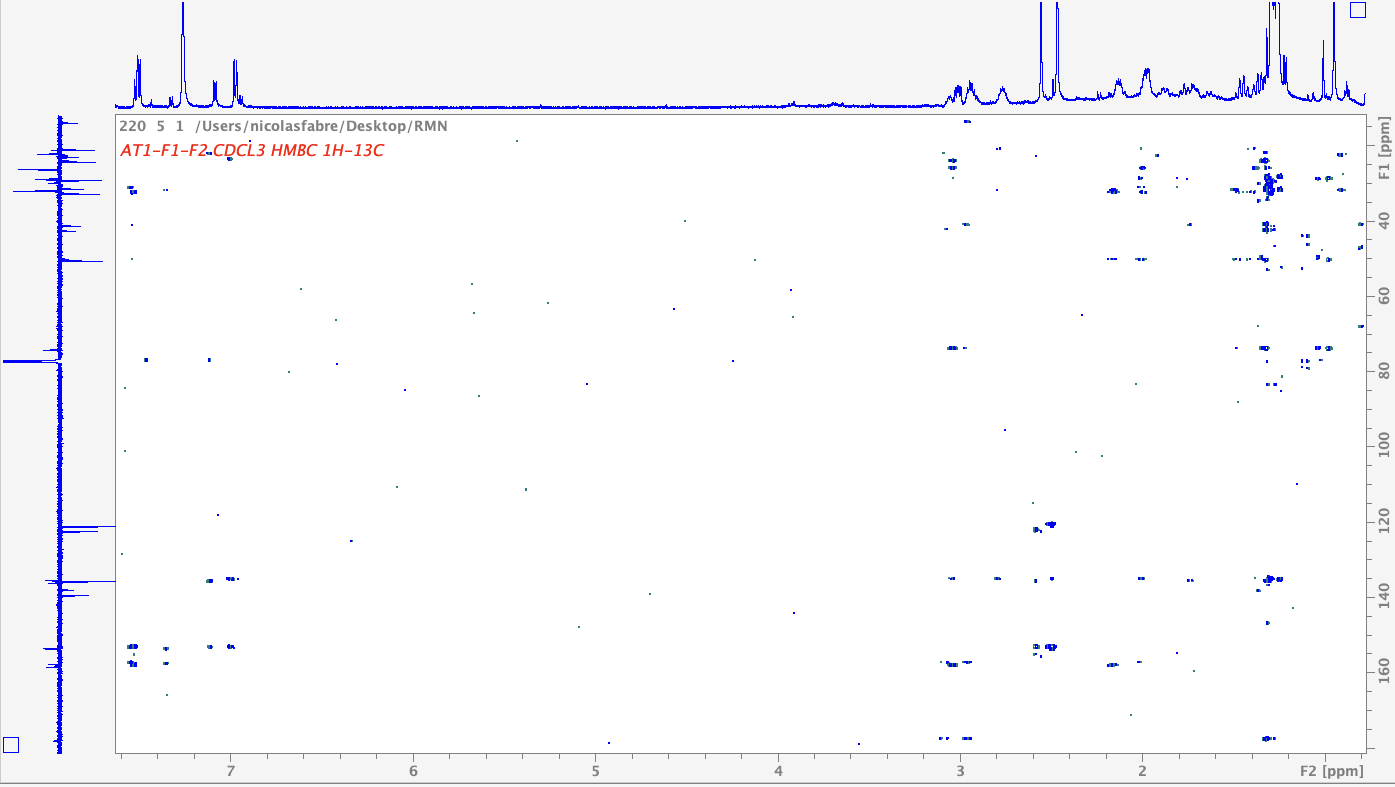


**Fig S19:** HMBC NMR spectrum of compound **3** (500 MHz, CDCl_3_).


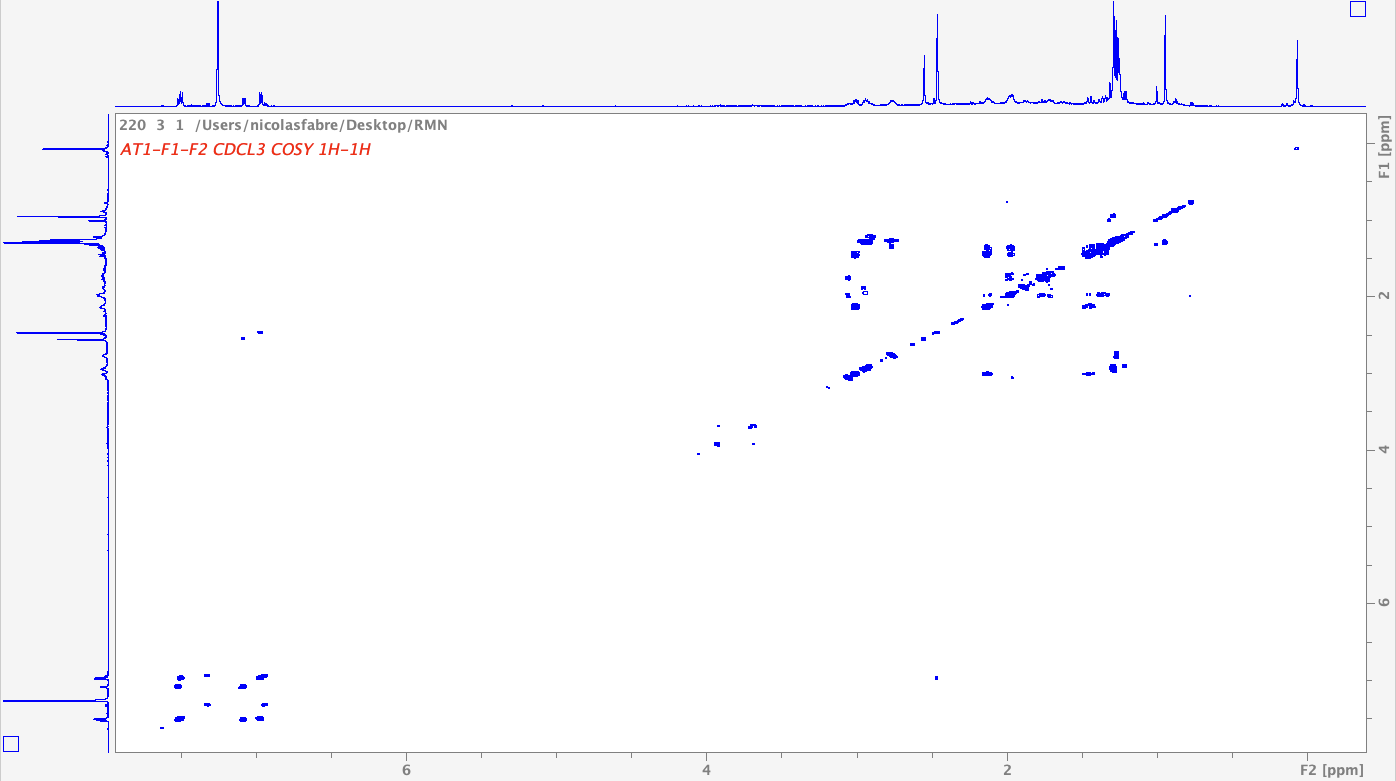


**Fig S20:** COSY NMR spectrum of compound **3** (500 MHz, CDCl_3_).


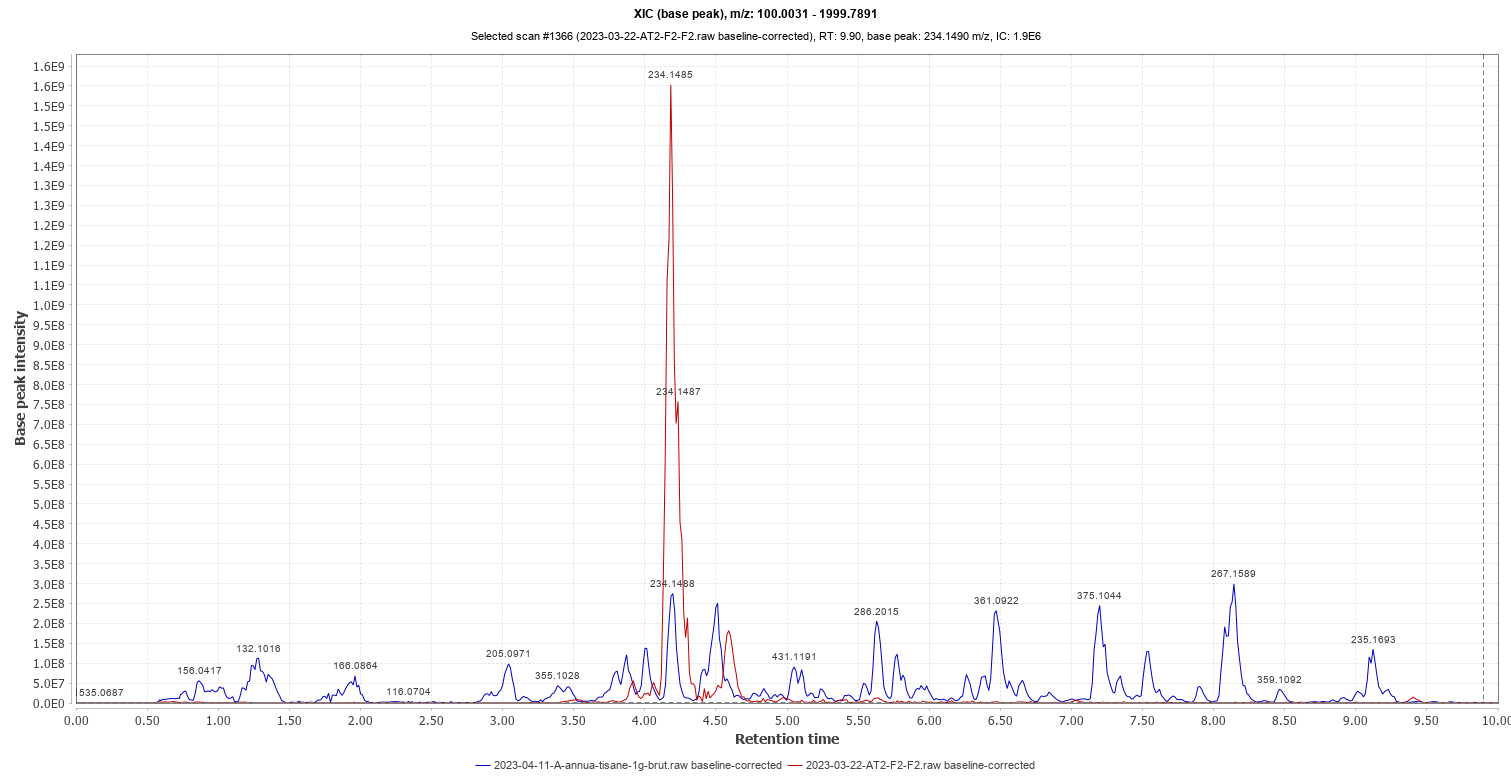


**Fig S21:** Comparison of the UPLC-HRMS chromatograms obtained for the *A. annua* tea (blue) and compound **2** (red).

Chromatograms were recorded under the following conditions: The column used was an Acquity PREMIER BEH C18 (100 x 2.1 mm i.d., 1.7 µm, Waters, Milford, MA, USA) column equipped with a guard column. Mobile phase A consisted of acetonitrile with 0.1% formic acid and mobile phase B was water with 0.1% of formic acid. The binary linear solvent gradient was as follows: At 0 min, 5% A-95% B; 0.5 min, 5% A-95% B; 10 min, 95% A-5% B; 12 min, 95% A-5% B; 12.5 min, 5% A-95% B; 15 min, 5% A-95% B. The flow rate was 0.3 mL/min, the column temperature was set to 40 °C.

| IC_50_ and CC_50_ in μM | Annuanine A (**1**) | Annuanine B (**2**) | Chloroquine | Artemisinin | Doxorubicin |
| --- | --- | --- | --- | --- | --- |
| *P. falciparum* FcB1 | > 429 | > 427 | 0.13 ± 0,011 | 0.081 ± 0,008 |  |
| Caco-2 cells | > 215 | > 214 |  |  | 1.98 ± 0,17 |
| Vero cells | > 215 | > 214 |  |  | 8.85 ± 0,43 |
| Thp1 monocytes | > 215 | > 214 |  |  | 0.79 ± 0,08 |

**Table S1**: IC_50_ (*P. falciparum*) and CC_50_ (other cells) of annuanine A, annuanine B, and positive controls, in μM.

**Fig S22**: Experimental graphs used for the determination of IC_50_ of annuanines A and B and controls against *P. falciparum* FcB1 (the concentrations on the X-axis are in μg/mL for annuanines A and B and in ng/mL for artemisinin and chloroquine).


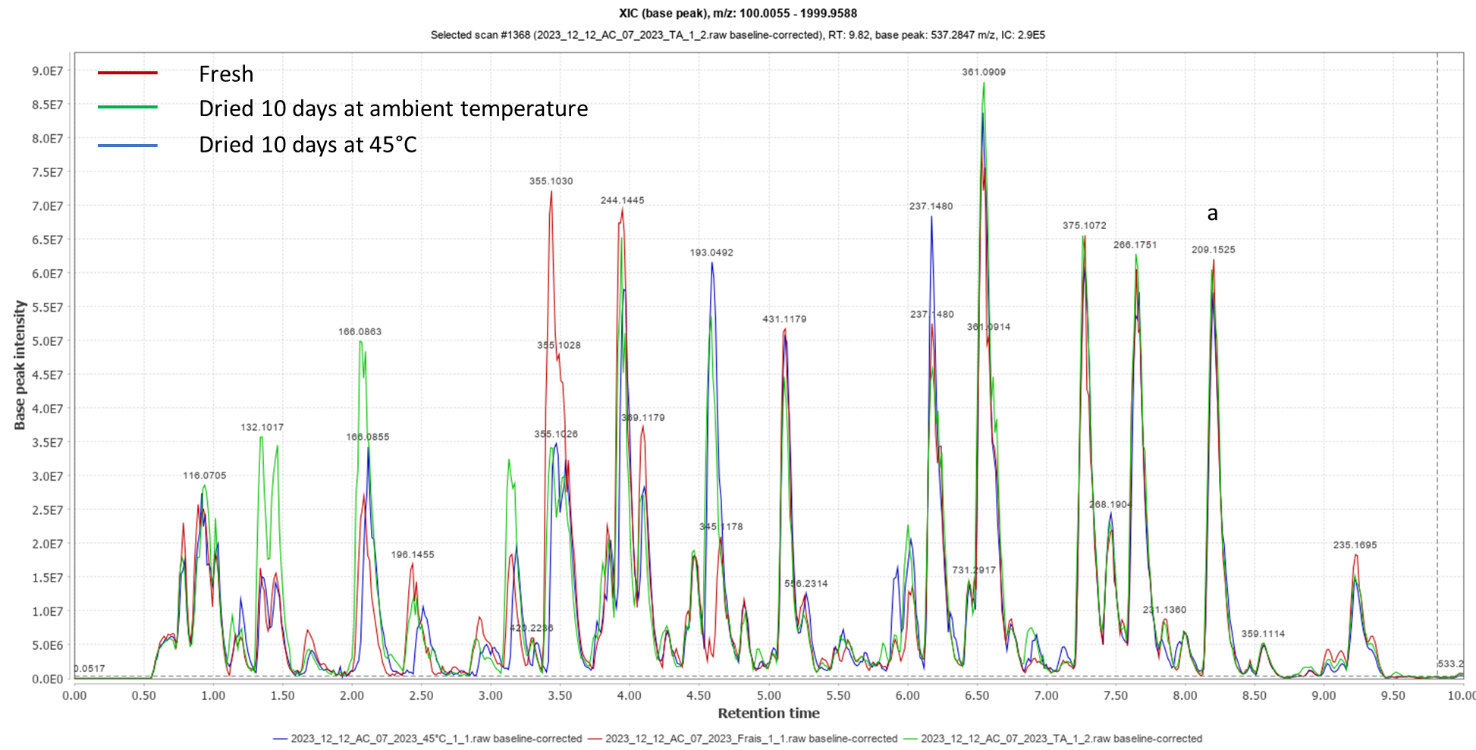


**Fig S23**: Whole LC-HRMS chromatogram (basepeak) of *A. annua* tea prepared with fresh plant, plant dried 10 days at ambient temperature, and plant dried ten days at 45°C. Peak **a** is the artemisinin peak. No signal corresponding to compound **2** (Rt 4.3 min) can be seen on the chromatograms, because of the low quantity of this compound.


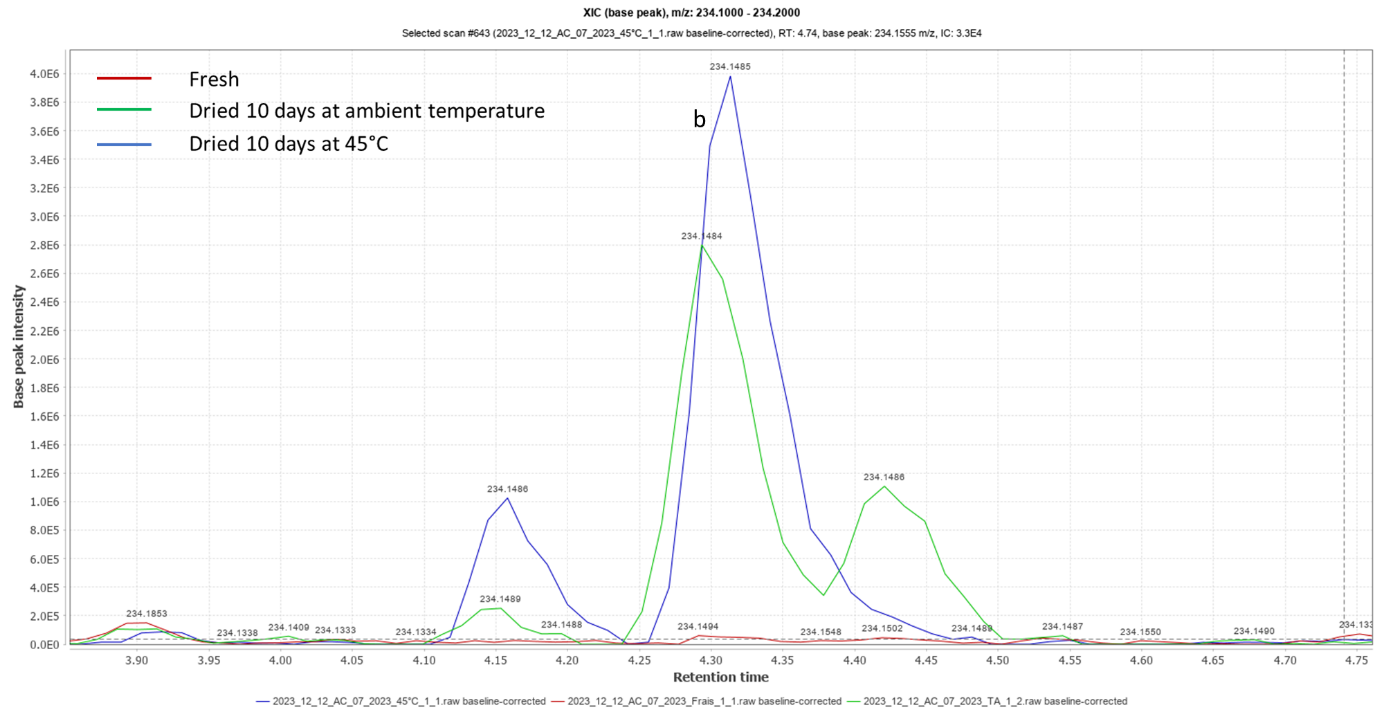


**Fig S24**: Extracted ion (*m/z* 234.1-234.2 = annuanine B) LC-HRMS chromatograms of *A. annua* tea prepared with fresh plant, plant dried 10 days at ambient temperature, and plant dried ten days at 45°C. Peak **b** corresponds to the signal of annuanine B.
