# Supplementary material for: Cadinapyridine sesquiterpene alkaloids from Artemisia annua and in vitro cytotoxicity and antiplasmodial activities
Source: PLoS One. 2025 Sep 3;20(9):e0331186. doi: 10.1371/journal.pone.0331186 (PMC12407398; doi:10.1371/journal.pone.0331186)
Supplement: Attachment — Submitted filename: Fabre et al .docx [file pone.0331186.s002.docx]

Review on the article: Cadinapyridien sesquiterpene alkaloids from Artemisa annua L.

By Fabre et al,

Generally, the article describes new structures (molecules found in A.
annua). Although they did not show antiplasmodial activity neither
activity against abnormal cells (perhaps cancerous cell), they could
contribute on the chemistry of A. annua.
However, the manuscript is poorly written, the biological tests
(antiplasmodial, anticancer, MTT assay) are poorly described with lack
of references in most cases. The authors talk about A. annua teas
throughput but the compounds isolated are not from the teas. Theres no
coherence, lack of consistency in the methodology and results. The
conclusion is misleading (not clear).
The results section is poorly described with no information on the
biological test.
I therefore recommend the manuscript to be reformatted, and rewritten
to focus on phytochemistry of A. annua and lay emphasis on the new
isolated molecules.
