# Supplementary material for: Cadinapyridine sesquiterpene alkaloids from Artemisia annua and in vitro cytotoxicity and antiplasmodial activities
Source: PLoS One. 2025 Sep 3;20(9):e0331186. doi: 10.1371/journal.pone.0331186 (PMC12407398; doi:10.1371/journal.pone.0331186)
Supplement: Attachment — Submitted filename: Supplementary Materials. review.docx [file pone.0331186.s004.docx]

**Supplementary Materials**

**Cadinapyridine sesquiterpene alkaloids from *Artemisia annua***

Inlcude the three structures in this section (Compound 1, 2 and 3)

Also include the table of the results of the activities with the concetrations used and the graphs of the IC_50_

**Fig S1:** Positive ion MS and MS/MS spectra of compound **1**.

**Fig S2:** ^1^H NMR spectrum (strength of magnet: 500 MHz or, solvent) of compound **1**.

**Fig S3:** ^13^C NMR spectrum (strength of magnet: 500 MHz or, solvent) of compound **1**.

**Fig S4:** HSQC NMR spectrum (strength of magnet: 500 MHz, solvent) of compound **1**.

**Fig S5:** HMBC NMR spectrum (strength of magnet: 500 MHz or, solvent) of compound **1**.

**Fig S6:** COSY NMR spectrum (strength of magnet: 500 MHz or, solvent) of compound **1**.

**Fig S7:** NOESY NMR spectrum (strength of magnet: 500 MHz or, solvent) of compound **1**.

**Fig S8:** Positive ion MS and MS/MS spectra of compound **2**.

**Fig S9:** ^1^H NMR spectrum (strength of magnet: 500 MHz or, solvent) of compound **2**.

**Fig S10:** ^13^C NMR spectrum (strength of magnet: 500 MHz or, solvent) of compound **2**.

**Fig S11:** HSQC NMR spectrum (strength of magnet: 500 MHz or, solvent) of compound **2**.

**Fig S12:** HMBC NMR spectrum (strength of magnet: 500 MHz or, solvent) of compound **2**.

**Fig S13:** COSY NMR spectrum(strength of magnet: 500 MHz or, solvent) of compound **2**.

**Fig S14:** NOESY NMR spectrum (strength of magnet: 500 MHz or, solvent) of compound **2**.

**Fig S15:** Positive ion MS and MS/MS spectra of compound **3**.

**Fig S16:** ^1^H NMR spectrum (strength of magnet: 500 MHz or, solvent) of compound **3**.

**Fig S17:** ^13^C NMR spectrum (strength of magnet: 500 MHz or, solvent) of compound **3**.

**Fig S18:** HSQC NMR spectrum (strength of magnet: 500 MHz or, solvent) of compound **3**.

**Fig S19:** HMBC NMR spectrum (strength of magnet: 500 MHz or, solvent) of compound **3**.

**Fig S20:** COSY NMR spectrum (strength of magnet: 500 MHz or, solvent) of compound **3**.

**Fig S21:** Comparison of the UPLC-HRMS chromatograms obtained for the *A. annua* tea (blue) and compound **2** (red).

Chromatograms were recorded under the following conditions: The column used was an Acquity PREMIER BEH C18 (100 x 2.1 mm i.d., 1.7 *µ*m, Waters, Milford, MA, USA), equipped with a guard column. Mobile phase A consisted of acetonitrile with 0.1% formic acid and mobile phase B was water with 0.1% formic acid. The binary linear solvent gradient was as follows: At 0 min, 5% A-95% B; 0.5 min, 5% A-95% B; 10 min, 95% A-5% B; 12 min, 95% A-5% B; 12.5 min, 5% A-95% B; 15 min, 5% A-95% B. The flow rate was 0.3 mL/min, the column temperature was set to 40 °C.
