# Supplementary material for: Cadinapyridine sesquiterpene alkaloids from Artemisia annua and in vitro cytotoxicity and antiplasmodial activities
Source: PLoS One. 2025 Sep 3;20(9):e0331186. doi: 10.1371/journal.pone.0331186 (PMC12407398; doi:10.1371/journal.pone.0331186)
Supplement: Attachment — Submitted filename: Some comments and suggesions.docx [file pone.0331186.s005.docx]

**Overall Summary:**

The manuscript describes the isolation and structural elucidation of two novel cadinapyridine sesquiterpene alkaloids (1 and 2) and the known compound fabianine (3) from **Artemisia annua** L. The compounds were extracted using LC-MS metabolomic profiling, and their structures were determined through NMR, MS, and X-ray crystallography. Despite lacking in vitro antimalarial or cytotoxic activity, this study enhances the chemical diversity of **A. annua** and suggests a plausible biosynthetic pathway related to artemisinin degradation.

The research is methodologically sound and fits well within the scope of PLOS ONE. However, some improvements could clarify and strengthen the manuscript's impact.

**Major Comments:**

1	The biological testing included Caco-2, Vero, Thp1 cells, and *Plasmodium falciparum.* While this is standard, there is no clear rationale for selecting these particular cell lines. Provide a brief justification in the Discussion section for why these specific models were chosen (e.g., relevance to toxicity screening, immune response, cancer biology, etc.).

2	None of the tested compounds showed significant cytotoxicity or antimalarial activity, yet this result is not thoroughly discussed. Discuss potential reasons for the lack of activity:

3	There is a suggestion that compounds may be degradation products of artemisinin during storage, but this is not fully substantiated. Expand on how the stability of the compounds was ensured during extraction and analysis. Were any controls performed to test compound formation under different storage conditions? This would strengthen the hypothesis regarding degradation origins.

4	Finally, the structural novelty of compounds **1** and **2** is emphasized, but limited comparisons are made with other known sesquiterpene alkaloids. Thoroughly discuss the Spectra data in details

**Minor Comments:**

1	The abstract states that compounds 1 and 2 were evaluated for growth inhibition but does not mention that they were inactive.

2	The preparation of teas used in the metabolomic analysis is only briefly mentioned.

3	There is some repetition in the introduction about the history and use of *Artemisia annua*.

4	The term “cadinapyridine” is proposed, but its usage should be consistent throughout the manuscript. Define the term early in the discussion and ensure it is used consistently in both text and figure captions.

5	The presentation of NMR data could be improved for readability. Use tables consistently for all compounds rather than mixing tabular and text formats.

**Recommendation:**

Accept with Revisions: This manuscript makes a valuable contribution to the phytochemical knowledge of *Artemisia annua*, particularly in identifying rare alkaloids. With minor revisions and clarification of key points, it will be suitable for publication in *PLOS ONE*.

Reviewed by Ivan Kiganda

Consider other corrections in the Manuscript text and the supporting infromation
