# Supplementary material for: Cadinapyridine sesquiterpene alkaloids from Artemisia annua and in vitro cytotoxicity and antiplasmodial activities
Source: PLoS One. 2025 Sep 3;20(9):e0331186. doi: 10.1371/journal.pone.0331186 (PMC12407398; doi:10.1371/journal.pone.0331186)
Supplement: Attachment — Submitted filename: Response to Academic Editor and Reviewers.docx [file pone.0331186.s008.docx]

**Responses to Academic Editor and Reviewers**

First of all, on behalf of all the co-authors, I would like to thank the academic editor and all the reviewers for their thorough work in reviewing our manuscript. As a regular reviewer myself, I understand the importance of this laborious and often undervalued work. That is why we will strive to provide detailed responses to all your questions and comments.

In order to clarify some legitimate misunderstandings, it is important to mention that when we submitted this article to PLOS ONE, we specified in the cover letter that the molecules discussed in this manuscript were highlighted during a metabolomic study conducted by UHPLC-MS on different herbal teas (traditional preparations) obtained from various samples of Artemisia annua collected in Benin and France with the aim to investigate the antimalarial efficacy of traditional preparations. We thought (perhaps mistakenly) that it would be interesting to submit these two studies simultaneously to PLOS One. Thus, another manuscript titled “Comparison of the composition and antiplasmodial activity of Artemisia annua teas using an untargeted metabolomic approach” was submitted under the number PONE-D-25-20883 and is currently under favorable revision. We will strive to be clearer in the revised manuscript to avoid some misunderstandings. However, the revision of the article dealing with the metabolomics of Artemisia teas was submitted the 29^th^ of June, and we hope that, if it is accepted, we will be able to cite it as reference in the present article by the end of the review process.

1. **Response to Academic Editor**

**Title: This title does not indicate if any bioactivity were assessed**

**Response**: it was modified for “Cadinapyridine sesquiterpene alkaloids from *Artemisia annua* and *in vitro* cytotoxicity and antimalarial activities”.

**Abstract**

**L18: How about Europe? It will make sense to say something about it since the samples were from Benin and France**

**Response**: Artemisia annua has no traditional use in Europe, and its use is not authorized. However, it grows in Europe, and in the metabolomic study discussed in the other article, we aimed to compare its chemistry with that of African samples.

**L20: define LC-MS**

**Response**: done

**L19-22: But I see Caco-2 cells which are cancer cells in your study**

**Response:** Indeed, the lines 19-22 were referring to our previous work on antimalarial tea. In the present work, Caco2 cells were used to assess the potential cytotoxicity of the new compounds characterized from *A. annua*, in addition to the evaluation of their antiplasmodial activity. To be clearer, the sentence: “To investigate the antimalarial efficacy of these traditional preparations, a LC-MS metabolomic approach was conducted on various teas prepared from different batches of *A. annua* collected in Benin and France, highlighting an original nitrogenous compound” has been changed to the following: “In another work, our team investigated the antiplasmodial efficacy of these traditional preparations using a Liquid-Chromatography-Mass Spectrometry metabolomic approach on various teas prepared from different batches of *A. annua* and highlighted an original nitrogenous compound. The present work aimed to isolate and characterize its structure”.

**L25**: Yes, MS and not liquid chromatography coupled to mass spectrometry

**L53-55: Too long, and not coordinating. Please rephrase**

**Response**: The sentence “These constituents can be divided into aliphatic hydrocarbon derivatives with varying levels of oxidation, aromatic hydrocarbons such as phenylpropanoids, and flavonoids, which constitute the second largest group after terpenoids” has been changed to the following: “These constituents can be categorized into three main groups: terpenoids with varying levels of oxidation, aromatic hydrocarbons like phenylpropanoids, and flavonoids. The latter group constitutes the second largest category after terpenoids. »

**L58: Please reduce on this style of writing. Putting words in parentheses will only but distort the communication being made**

**Response**: this has been done

**L63-71**: **This is not what you indicated in the abstract. For this section, only indicate the aim of the study. The INTRODUCTION should not already tell us all the methods and results obtained thereof**

**Response**: the last paragraph of INTRODUCTION has been changed as follows: “A recent study conducted by our team focused on investigating the antimalarial efficacy of traditional preparations of Chinese wormwood using an untargeted metabolomic approach conducted on various teas prepared from different batches of *A. annua* collected in Benin and France. The phytochemical compositions, combined with *in vitro* IC_50_ values on *Plasmodium falciparum* of different tea samples, revealed a feature with an original molecular formula, which appears to be linked to the best antimalarial activity. In the present work, we describe the structural elucidation and preliminary biological properties of this new derivative, along with two other structurally related compounds, including a known derivative. The biosynthetic origin of these secondary metabolites is also discussed.

**Materials and Methods**

**L89: Aerial parts?**

**Response**: Yes, but without flowers, which is why we mention stems and leaves. The term “aerial parts” can imply flowers.

**L92: where is the sample from France?**

**Response**: The sample from France was used for the other study (the metabolomic study). For this work, only Beninese sample was used.

**L97: Ammonium hydroxide 28-30%, why percentage range?**

**Response**: This means that there are between 28 g and 30 g of NH_3_ in 100 mL of water to form 28-30% NH_4_OH. It is a classic reagent in chemistry.

**L333: Do not mix freely NMR interpretation with MS data**

**Response:** The delta difference of 1.6 ppm is an MS term representing the high-resolution difference between the measured mass and the calculated mass, which is therefore 1.6 millionths, making the proposed molecular formula very reliable.

**L337: m/z in italics**

**Response**: all m/z in the text have been italicized.

**L399**: the term ‘comparable’ is indeed more appropriate than ‘same’.

**L408**: **If this term was already used before, then why are you proposing it for the current compounds? I would simply identify them as such if they meet the criteria/skeleton proposed/specified by previous authors.**

**Response**: The term cadinane pyridine sesquiterpene is used here for the first time, as indicated in lines 375 and 376. We propose to name this new family of sesquiterpenes ‘cadinapyridines’ by analogy with gaipyridines sesquiterpenes biogenetically formed from a gaiane skeleton. Compounds **1** and **2** are the second and third representatives of this kind of sesquiterpenes, after fabianine, and in articles describing fabianine no discussion regarding the construction and originality of its structure was provided. Cadinapyridine is a new name proposed by us.

**L408**: Institut de chimie has been translated by ‘Toulouse Chemistry Institute’.

2. **Response to reviewer 1.**

**Generally, the article describes new structures (molecules found in A. annua). Although they did not show antiplasmodial activity neither activity against abnormal cells (perhaps cancerous cell), they could contribute on the chemistry of A. annua. However, the manuscript is poorly written, the biological tests (antiplasmodial, anticancer, MTT assay) are poorly described with lack of references in most cases.**

**Response:** The manuscript has been thoroughly revised in response to the questions and comments from the other reviewers and the academic editor. References have been added for the biological tests, and a summary table of the biological results has been included in the supplementary materials (S1 Table).

**The authors talk about A. annua teas throughput but the compounds isolated are not from the teas. Theres no coherence, lack of consistency in the methodology and results. The conclusion is misleading (not clear). The results section is poorly described with no information on the biological test. I therefore recommend the manuscript to be reformatted, and rewritten to focus on phytochemistry of A. annua and lay emphasis on the new isolated molecules.**

**Response**: The confusion regarding the Artemisia teas is explained in the introduction of this response letter. The study of the teas is the subject of another article submitted to the same journal, which has just undergone revision. We should be able to reference it (if this article is accepted) by the end of the review process for the current article. We agree that discussing the teas was not at all clear and have modified the article to avoid any legitimate confusion. The results section has been merged with the discussion to produce a clearer and more coherent text. The conclusion has been completely rewritten. We believe that all these modifications have significantly improved the clarity and quality of our article.

3. **Response to reviewer 2.**

**Major Comments:**

1 **The biological testing included Caco-2, Vero, Thp1 cells, and *Plasmodium falciparum.* While this is standard, there is no clear rationale for selecting these particular cell lines. Provide a brief justification in the Discussion section for why these specific models were chosen (e.g., relevance to toxicity screening, immune response, cancer biology, etc.).**

**Response:** in order to be clearer, the sentence:

‘As compound **2** was first highlighted in *A. annua* teas following a metabolomic analysis of several samples revealing significant differences in their *in vitro* antimalarial activities, compounds **1** and **2** were evaluated for cell-growth inhibition on one cancer cell line (human colorectal adenocarcinoma Caco-2) and two healthy cell lines (monkey kidney epithelial Vero and human monocytes Thp1), as well as on the *Plasmodium falciparum* FcB1 strain’ has been modified as follows:

‘Since compound **2** was initially identified in A. annua teas through a metabolomic analysis of various samples, which revealed significant differences in their *in vitro* antimalarial activities, compounds 1 and 2 were assessed for their bioactivities against the chloroquine-resistant Plasmodium falciparum FcB1 strain. To evaluate potential selectivity for activity against P. falciparum or cancer cells, both compounds were also tested for their ability to inhibit cell growth in human and non-human healthy cells (human monocytes Thp1 and monkey kidney epithelial Vero and), as well as in a cancer cell line (human colorectal adenocarcinoma Caco-2).’

2 **None of the tested compounds showed significant cytotoxicity or antimalarial activity, yet this result is not thoroughly discussed. Discuss potential reasons for the lack of activity:**

**Response**: We were indeed very surprised to find no effect on Plasmodium and the tested cell lines. As stated in the article, we hypothesized a potential synergistic effect of compound **2** with artemisinin, and therefore conducted tests on Plasmodium by mixing the two molecules, but it turned out that only artemisinin is active. It would indeed be interesting to discuss this lack of effect on the tested cell lines, but we could not find any biological data for related analogs in the literature. The only natural compound representative of this family is fabianine and, as we mentioned in the publication, **2** was already mentioned as a synthetic compound, but nothing is reported in the literature regarding their biological activity. Thus, we think it is complicated to have a discussion without being able to rely on already published facts, risking talking in vain.

3 **There is a suggestion that compounds may be degradation products of artemisinin during storage, but this is not fully substantiated. Expand on how the stability of the compounds was ensured during extraction and analysis. Were any controls performed to test compound formation under different storage conditions? This would strengthen the hypothesis regarding degradation origins.**

**Response**: Due to the experimental condition of alkaloid extraction, we cannot rule out that artemisinin (or another molecule present in *A. annua*) was transformed in compounds **1** and **2** during the extraction process. Indeed, compound **1** was not detected in *A. annua* teas and maybe a purification artifact. Nevertheless, its crystallization and the obtention of its X-Ray structure was necessary to establish the structure of compound **2** with certainty, especially for the 3D-structure.

Compound **2** was much more interesting for us, because it displayed the same retention time, MS and MS/MS spectra as the compound detected in the tea and whose identification was the aim of this work. So, we conclude with confidence that the alkaloid present in the tea is compound **2**.

In our precedent work, we prepared two series of teas from dried and powdered *A. annua* harvested in 10 plantations in Benin. The first series was prepared three months after harvesting, drying and grinding the plant. The second series is prepared fifteen months after. A metabolomic study comparing the two series revealed that the most discriminant feature between them was signal of compound **2**, which was higher in series 2. That why we make the hypothesis that compound **2** maybe a degradation product appearing during the conservation of the dry crushed plant.

We also present here additional data showing that compound **2** signal was absent in a tea made from fresh *A. annua*, and started to appear after the drying process (Fig S23 and Fig S24).

We explained this in the “biosynthetic origin” section, between lines 384 and 400 of the revised version of the text.

4 **Finally, the structural novelty of compounds 1 and 2 is emphasized, but limited comparisons are made with other known sesquiterpene alkaloids. Thoroughly discuss the Spectra data in details**

**Response**: It is mentioned in the discussion that compounds **1** and **2** are the second and third representatives of this structural group, featuring a cadinane-type skeleton with a pyridine core, while the closest structures already published exhibit a guaiane skeleton with a pyridine core (the guaipyridines known as rupestines). It is indeed possible to propose an easy way to differentiate them from a spectral data perspective: guaipyridines contain 15 carbons in their basic structure, whereas cadinapyridines have only 14. The sentence: ‘The two structural groups can be easily differentiated based on their ^13^C NMR spectra, as guaipyridines contain fifteen carbons in their basic structure, whereas cadinapyridines have only fourteen’ has been added in this regard in the discussion.

**Minor Comments:**

1 **The abstract states that compounds 1 and 2 were evaluated for growth inhibition but does not mention that they were inactive.**

**Response**: the penultimate sentence of the abstract clarifies this fact and was rephrased: ‘Unfortunately, the two compounds **1** and **2** were inactive in these *in vitro* models.’

2 **The preparation of teas used in the metabolomic analysis is only briefly mentioned.**

**Response**: This is because the Artemisia teas were not used in this study but in the metabolomic analysis we refer to, which is another study conducted within our team (see the explanation in the introduction to this response document).

3 **There is some repetition in the introduction about the history and use of *Artemisia annua*.**

**Response**: The set of corrections proposed by the reviewers and the academic editor has improved this point.

4 **The term “cadinapyridine” is proposed, but its usage should be consistent throughout the manuscript. Define the term early in the discussion and ensure it is used consistently in both text and figure captions.**

**Response**: The term cadinapyridine is introduced at the beginning of the discussion, on the sixth line out of a total of 50 lines. This term could indeed replace the term 'compound' in certain parts of the text or the legends (for example, 'structure of cadinapyridines **1**-**3**'), but we are concerned that overusing this term might confuse the reader. Consequently, we prefer to use it only in the legends for Figures 1 and 4 (Structures of cadinapyridines **1**-**3**/^1^H-NMR multiplicities of H-7, H-10 and H-11 for cadinapyridines 1-**3**).

5 **The presentation of NMR data could be improved for readability. Use tables consistently for all compounds rather than mixing tabular and text formats.**

**Response**: This type of presentation is the classic format for demonstrating the structure of new natural compounds. The NMR data for all three compounds are compiled in a single table, and the text details their use for the developed structure demonstration. We could add the 2D NMR data to the table, but this might make it too cumbersome, so we chose to present them in a figure (Fig 2) showing the main correlations.

**Responses to annotations throughout the manuscript:**

**What is L. in the title? write in full**

**Ensure that the title of the manuscript matches that of the supporting information, and also include the list of authors in the supporting information.**

**Response**: L. means Linné, the official abbreviation of the botanist who first described this species in the 18^ht^ century. The title has been modified according to the recommendation of academic editor. Regarding the format of the supporting information file, we have complied with the journal's format and the authors' guidelines.

**L31-32 Rephrase this sentence 31 and 32 since you earlier suggested that the compounds were inactive.**

**Response**: This sentence does not discuss activity but only chemistry

**L44 Be consistent with the authority of the species it should be uniform.**

**Response**: It is customary, once a species has been cited in full, to cite it in abbreviated format.

**L59 Should be italicized**

**Response**: yes, it has been modified throughout the manuscript

**L60 Remove this comma**

**Response**: done

**L62: Is it only or two derivatives as you have compound 1 and 2 new**

**Response**: 2 new compounds since in the sentence it is specified that there is 1 known compound (fabianine), implying a total of three (and so, two are new). We put emphasis on compound **2** as it is the compound which was highlighted in our previous study on *A. annua* teas. Compound **1** was isolated in this study, but was not present in the *A. annua* teas, as we mentioned in lines 424-425 of the revised manuscript.

**L93: a space:**

**Response**: done

**L120: This mode part should be described in the General material section**

**Response**: done at the beginning of the general material section

**L123**: done

**L160**: done

**L204: µ italicise all these ones**

**Response**: Units should not be italicized.

**L255: You should also discuss some key HMBC correlations in the connectivity of the structure.**

**Response**: We believe that Figure 3 is sufficiently clear, especially since the structure is confirmed by X-ray diffraction.

**L266: It would be better to list them and also suggesting the reference for this sentence.**

**Response**: this has been done

**L269: it should be δH for proton resonance or δC for carbon resonance. H and C should be subscripts but δ should italicised**

**Response**: This has been done whenever necessary, but both ways of citing NMR data are possible.

**L272: Rephrase this sentence " three methine proton signals....**

**Response**: the sentence has been rephrased

**L278: since they are new skeleton it would be better to include the HMBC correlations in the table for compound 1 and 2**

**Response**: As mentioned above, we believe that including the HMBC data in the table would make it cluttered. We think a figure is much clearer.

**L278: Exactly which solvent did you use CDCl3 or CD2Cl2**

**Response**: All NMR experiments were recorded in CDCl_3_. It turns out that for compound **2**, some ^1^H NMR signals were overlapping, and hindered structural determination. Therefore, we recorded a spectrum in CD_2_Cl_2_, which allowed us to separate the signals of interest. So, for compound **2** in table 1, some key ^1^H NMR signals recorded in CD_2_Cl_2_ are also mentioned. They are noted with an asterisk referring to a footnote in the table to inform the reader. This comparison between ^1^H NMR experiment recorded in CDCl_3_ and in CD_2_Cl_2_ is also depicted for compound **2** in the figure 4 . To make thinks clearer, we changed the position of the asterisk and the footnote of the table 1 “* Recorded in CD_2_Cl_2_” for “*Signal obtained when the spectra are recorded in CD_2_Cl_2_”

**L295: Please suggest the IUPAC name for this compound also you can give the trivial name**

**Response**: IUPAC names have been indicated for compounds **1**-**2** together with the trivial names annuanines A and B.

**L322: it should be δH for proton resonance or δC for carbon resonance. H and C should be subscripts but δ should italicised**

**Response**: this has been done

**L328: Please suggest the IUPAC name for this compound also you can give the trivial name**

**Response**: done

**How do you know and yet you suggested that they have not been discussed before**

**Response**: A thorough search in databases such as the Dictionary of Natural Products and CAS SciFinder confirmed the novelty of these compounds as natural compounds. As we mentioned lines 402-406 of revised manuscript, compound **2** was already synthetized as an intermediate in the synthesis of a Smo antagonist (Liu et al. J. Med. Chem. 2016, DOI: 10.1021/acs.jmedchem.6b01247). In the cited publication, it was not isolated nor characterized by mass spectrometry or NMR. Its name was not mentioned neither.

**L384: None of the tested compounds showed significant cytotoxicity or antimalarial activity, yet this result is not thoroughly discussed. Also include the table of results in the supporting information. Discuss potential reasons for the lack of activity. Why was compound 3 not tested**

**Response**: as mentioned above the only known representative of this compound family is fabianine, and nothing is reported in the literature regarding its biological activity. Thus, we think it is complicated to have a discussion without being able to rely on already published facts, risking talking in vain. A table of biological results have been introduced in supporting information. Fabianine was not biologically tested due to its low quantity (less than 2 mg) and lack of stability (the poor quality of the NMR spectra can be seen in supporting information).

**Comments concerning the supporting materials file**

**Title: Let it be the same as that of the title of the manuscript and also include the authors.**

**Response**: The title is now the same and authors and affiliations have been included.

**Include the three structures in this section.**

**Response:** the structures of each compound have been included inside the corresponding ^1^H NMR spectrum.

**Include the table of the results of the activities with the concentrations used and the graphs of the IC_50_.**

**Response**: This has been done

We have taken into account all the other requested modifications (strength of magnet and solvent, etc.).

4. **Response to reviewer 3.**

**Abstract: The authors need to name the new compounds 1 and 2 in the abstract, since this is their main finding.**

**Response**: done. The proposed names annuanine A and annuanine B have been introduced in the abstract.

**Introduction: The introduction starts well with the description of the plant, its uses, the studies that have been done on the plant and the description of the identified phytochemicals. Then the chapter ends with a paragraph that highlights the aim, methods and partial findings, which sounds more of an abstract.**

**Comment: this chapter misses the research gap, and the specific research questions to be addressed.**

**Response**: We completely agree with this comment because, due to a lack of clarity on our part, it was difficult to understand that the metabolomic study that revealed the compounds of interest described in this article was the subject of another article produced by our team and submitted simultaneously to PLOS One (as explained in the introduction of this response letter). The rephrasing of the penultimate sentence of the introduction clarifies that what is described there does not pertain to partial results and helps to understand what led to the research question posed: to identify these original molecules, evaluate their biological activity, and discuss the origin of this very particular structural skeleton.

**Materials and Methods**

**-Line 85: The subheading “General materials” is misleading, since it doesn’t seem related to what is under it. Kindly give the appropriate heading, and then shift it to be after the section**

**“Extraction and purification of alkaloids”. Because, purification comes after Extraction. See line 86 says “Compounds were purified on silica cartridges...” You can’t purify before extraction please. Have some logical flow of ideas.**

**Response**: The heading has been changed to “General analysis and purification equipment” and shifted after the section describing the purification of alkaloids.

**-Kindly maintain the same tense under this section. There is mixed use of “was” and “is”**

**Response**: Done

**-Line 132: How did you identify Fabianine (F1-4, compound 3) at this level, before LC-MS and NMR. Much as it is a known compound, it was not identified at this point. So, I would suggest just putting “compound 3 (F1-4),**

**Response:** We agree

**-Line 138. This coding of subfractions is confusing “compound 1 (F4-1 - 2 mg)” It could mean subfraction 1 of fraction 4 or whatever... What would it mean for someone who considers it as fraction F4-1 -2?? And then gets stuck at the meaning of mg? Does that become the problem of the reader or the writer? Check that all the codes are defined to avoid confusion.**

**Response**: We agree. To avoid any confusion, the amount of compound obtained has been removed from the parentheses and included in the sentence. Thus, for example, the sentence "compound 2 (F4-1, 2 mg) was obtained" becomes "2 mg of compound 2 (F4-1) were obtained."

**-Lines 147 to 156 look like results to me, yet there is a section of results. I would request the authors to have logical flow of information/ideas**

**Response**: These lines correspond to the description of the physicochemical data characterizing the molecules described, which have no relevance for demonstrating the developed structures outlined in the results section. It is customary in all articles describing new chemical structures to list these data in the experimental section. We believe it is advisable to adhere to this practice.

**Results
-Though this section highlights results, some discussions of the results appear here, only that the references are lacking. Phrases like “...indicating six degrees of unsaturation (Line 258)”, “...suggesting a primary amine (loss of NH3) in the structure of 1. (Line 260)”, “...indicating the presence of three methyl groups, two methylene groups, five methines (including two aromatics), and four quaternary carbons... (Lines 263 down wards...)”, “...were in good agreement with those of Fabianine [18], an alkaloid first isolated from Fabiana imbricata by Edwards and Elmore in 1962. [19] Therefore,... (Line 335 down wards...”
-How can a section of results have references even?
-I would think, interpretation of the findings or adding meaning to them is discussion.
-The authors need to cite the literature that guided the MS and NMR assignments
NB: The authors need to know what goes where and in which section.**

**Response**: We agree with this remark. We propose to merge the results and discussion sections into a single one divided into five subsections (structure of compound 1, structure of compound 2, structure of compound 3, biosynthetic origin, and biological evaluation). This allows for elements of discussion to be introduced throughout the section and to include references. For the NMR and MS references, two citations have been added to demonstrate the pyridine core. The other deductions (for example, the loss of 17 u in MS suggesting NH3) are evident to a specialist in the field and are normally not supported by a reference.

**Conclusion:
-The authors lack the research gap and their questions are not articulated. Thus, it is very hard to assess their conclusion, to see if it is matching**

**Response**: We agree with this remark. The conclusion has been completely rewritten as follows: « In this study, we isolated and characterized two previously undescribed cadinapyridine sesquiterpene alkaloids (annuanines A and B) from the leaves and stems of A. annua, along with the known alkaloid fabianine. The absolute configuration of annuanine A was confirmed by X-ray diffraction. Given the very rare chemical skeleton of these compounds, we proposed a biogenetic pathway indicating that these cadinapyridine alkaloids may originate from the degradation processes of artemisinin, highlighting the dynamic chemistry of A. annua during storage and preparation. Despite the structural originality of the isolated molecules, the biological evaluation showed that neither annuanine A nor B exhibited notable antimalarial activity against Plasmodium falciparum or cytotoxicity toward cancerous or healthy cell lines, raising questions about their pharmacological relevance. »
